# Genomic selection for resistance to spruce budworm in white spruce and relationships with growth and wood quality traits

**DOI:** 10.1111/eva.13076

**Published:** 2020-08-11

**Authors:** Jean Beaulieu, Simon Nadeau, Chen Ding, Jose M. Celedon, Aïda Azaiez, Carol Ritland, Jean‐Philippe Laverdière, Marie Deslauriers, Greg Adams, Michele Fullarton, Joerg Bohlmann, Patrick Lenz, Jean Bousquet

**Affiliations:** ^1^ Canada Research Chair in Forest Genomics Institute of Systems and Integrative Biology and Systems, and Centre for Forest Research Université Laval Québec QC Canada; ^2^ Natural Resources Canada Canadian Wood Fibre Centre Québec QC Canada; ^3^ Michael Smith Laboratories University of British Columbia Vancouver BC Canada; ^4^ Department of Forest and Conservation Sciences University of British Columbia Vancouver BC Canada; ^5^ J.D. Irving Limited Sussex NB Canada; ^6^ Forest Development Section Natural Resources and Energy Development Government of New Brunswick Island View NB Canada; ^7^ Department of Botany University of British Columbia Vancouver BC Canada; ^8^ Present address: Western Gulf Forest Tree Improvement Program Texas A&M Forest Service Forest Science Laboratory College Station TX USA

**Keywords:** acetophenone aglycones, acoustic velocity, genetic gain, *Picea glauca*, prediction accuracy, tree breeding

## Abstract

With climate change, the pressure on tree breeding to provide varieties with improved resilience to biotic and abiotic stress is increasing. As such, pest resistance is of high priority but has been neglected in most tree breeding programs, given the complexity of phenotyping for these traits and delays to assess mature trees. In addition, the existing genetic variation of resistance and its relationship with productivity should be better understood for their consideration in multitrait breeding. In this study, we evaluated the prospects for genetic improvement of the levels of acetophenone aglycones (AAs) in white spruce needles, which have been shown to be tightly linked to resistance to spruce budworm. Furthermore, we estimated the accuracy of genomic selection (GS) for these traits, allowing selection at a very early stage to accelerate breeding. A total of 1,516 progeny trees established on five sites and belonging to 136 full‐sib families from a mature breeding population in New Brunswick were measured for height growth and genotyped for 4,148 high‐quality SNPs belonging to as many genes along the white spruce genome. In addition, 598 trees were assessed for levels of AAs piceol and pungenol in needles, and 578 for wood stiffness. GS models were developed with the phenotyped trees and then applied to predict the trait values of unphenotyped trees. AAs were under moderate‐to‐high genetic control (*h* ^2^: 0.43–0.57) with null or marginally negative genetic correlations with other traits. The prediction accuracy of GS models (GBLUP) for AAs was high (*PAAC*: 0.63–0.67) and comparable or slightly higher than pedigree‐based (ABLUP) or BayesCπ models. We show that AA traits can be improved and that GS speeds up the selection of improved trees for insect resistance and for growth and wood quality traits. Various selection strategies were tested to optimize multitrait gains.

## 1 INTRODUCTION

Coevolution of herbivores and host plants has led to complex defense mechanisms with insect pests as an important selection force (Züst et al., 2012). The temporal and spatial presence or absence of herbivores in a sympatric area yields immediate ecological and evolutionary changes (Agrawal, Hastings, Johnson, Maron, & Salminen, 2012; Sork, Stowe, & Hochwender, 1993), which can create distinct geographic pattern of adaptive and resistance traits (Parent et al., 2017; Züst et al., 2012). This standing genetic variation in resistance to insect herbivory presents a unique opportunity for breeding programs in crops and trees to enhance resistance of planting stock, which will secure future crop or fiber yield and preserve plantation investments.

Over the last half century, tree breeding has been successful at providing many countries with genetically improved material for their reforestation programs (Mullin et al., 2011). Although it is possible to improve tree species simultaneously for several traits, the focus has generally been on growth traits. Other economically important traits, such as wood properties and insect resistance, are more costly and difficult to assess and require longer time frames. Despite its success, conventional tree breeding is intrinsically slow and takes decades before genetically improved stock is available for reforestation. For boreal conifers, it can take up to 30 years to complete a breeding cycle of recurrent selection of superior parent trees and testing their progeny. Moreover, the breeders’ paradigm until recently assumed that the climate and ecological site conditions were stable over time so that local seed sources were better adapted. Under this paradigm, it was possible to develop genetically improved material within breeding zones of stable climate conditions. However, this paradigm no longer holds with climate change. Over the long term, natural tree populations may have the potential for evolutionary response to climate change (Alberto et al., 2013). However, in the short term, tree breeders need more advanced methods and approaches to facilitate the simultaneous evaluation of a variety of ecologically and economically important traits in selected trees, and speed up the selection of superior trees. Such approaches are critical to maintain health and productivity of forest plantations.

Genomic selection (GS) was proposed by Meuwissen, Hayes, and Goddard (2001) as an alternative to conventional breeding based on pedigree selection and relies on genome‐wide distributed markers to model the entire complement of QTL effects across the genome to estimate genomic estimated breeding values (GEBVs) of individuals. It is an attractive approach because GS models combine genotypic and phenotypic data collected on individual in genetic tests that can be used to predict breeding values of genotyped, but unphenotyped, individuals or young seedlings without having to test them in the field. Moreover, GS models can be developed using a relatively small subset of individuals for traits that are cumbersome or expensive to assess in the field, such as pest or drought resistance. These models can then be used to predict the breeding values of a large numbers of unphenotyped candidates in order to make selections at high intensity. In forest trees, genomic breeding tools have been developed over the last decade that allow for accelerated screening and selection in large breeding populations and for combining different growth, wood quality, and resistance traits (Bartholomé et al., 2016; Beaulieu, Doerksen, MacKay, Rainville, & Bousquet, 2014; Grattapaglia & Resende, 2011; Lenz et al., 2017; Lenz, Nadeau, Mottet, et al., 2020; Li et al., 2019). Assessing resistance traits is becoming a pressing issue with new conditions of climate and environmental change. It follows that if GS is deployed economically on large number of candidates, multitrait selection combining growth, wood quality, and resistance to pest and other stress becomes more achievable than with conventional breeding (Lenz, Nadeau, Mottet, et al., 2020).

White spruce is a boreal conifer with a natural distribution that covers almost all of Canada and some northern states in the USA. It occurs on a variety of soils and under a wide range of climatic conditions (Farrar, 1995). It is also one of the most planted conifers in eastern Canada. Breeding programs have been in place over the last 50 years and have mainly focused on growth and adaptive traits (Mullin et al., 2011), which vary widely at the phenotypic level (Li, Beaulieu, Corriveau, & Bousquet, 1993; Nienstaedt, 1985). Over the last decades, emphasis has also been put on wood quality traits (Beaulieu et al., 2011; Corriveau, Beaulieu, & Daoust, 1991; Lenz, Cloutier, MacKay, & Beaulieu, 2010), because faster growth is generally negatively correlated with important wood quality traits such as density and mechanical properties (Corriveau et al., 1991; Ivkovich, Namkoong, & Koshy, 2002). Maintaining wood quality is thus important to meet the lumber grading construction standards.

Climate change is demanding white spruce breeding programs to accelerate breeding cycles to develop improved genetic material that will be adapted to future climatic conditions and better resistance to increasing occurrence of insect pests and diseases. Warmer temperatures, for instance, would lift the climate barriers to population growth or range expansion of native or invasive forest pests, potentially resulting in severe outbreaks (Gauthier, Bernier, Kuuluvainen, Shvidenko, & Schepaschenko, 2015). Insect outbreaks are already among the major natural disturbances that can cause widespread damages to natural forests and plantations. Spruce budworm (*Choristoneura fumiferana* (Clem), SBW) is a native insect prevalent in North Eastern America and a defoliator that periodically attacks spruce forests in eastern Canada (Blais, 1983; Dupont, Bélanger, & Bousquet, 1991; Gray & MacKinnon, 2006). It mainly feeds on balsam fir (*Abies balsamea* (L.) Mill.) and on spruces such as white spruce (*Picea glauca* (Moench) Voss) and to a lesser extent on black spruce (*Picea mariana* (Mill.) BSP). Molecular mechanisms of resistance of white spruce to SBW and its genetic control have recently been reported (Mageroy et al., 2015, 2017; Parent et al., 2017). Using a functional genomics approach, these authors reported that the expression levels and function of a β‐glucosidase gene, *Pgβglu‐1*, were underpinning natural resistance to SBW in mature white spruce trees. The encoded Pgβglu‐1 enzyme catalyzes the cleavage of acetophenone sugar conjugates, picein and pungenin, to release the corresponding acetophenone aglycones (AAs), piceol and pungenol (Mageroy et al., 2015). Pungenol and piceol aglycones commonly accumulate in spruce foliage in the form of the corresponding glycosides, pungenin and picein, but these glycosides appear to be inactive against the insect until they are cleaved by the β‐glucosidase Pgβglu‐1, which releases the active aglycones. These findings open the possibility to breed for resistance to SBW in spruces by selecting for elevated concentrations of the active aglycones in tested material.

Here, we present a proof‐of‐concept study of GS for enhancing resistance to defoliation by an herbivory insect and evaluating prospects for multitrait selection implicating resistance, growth, and wood quality. The objectives of the present study were to: (a) estimate the genetic control of AA concentrations related to SBW resistance in white spruce and better understand their genetic relationships with growth and wood quality traits; (b) evaluate the performance of genomic selection models for each of these traits; and (c) estimate the expected genetic gains for AAs alone and in conjunction with the other traits in various multitrait selection schemes.

## 2 MATERIALS AND METHODS

### 2.1 Genetic material and phenotyping

Data were collected in white spruce selection plantations established in New Brunswick, Canada, and managed by the New Brunswick Tree Improvement Council (NBTIC). These plantations regrouped full‐sib families that were produced by controlled pollinations between 212 elite parents (120 females and 118 males). Families were evaluated in 22 tests established on five sites in as many different localities (Table S1). For the present study, we sampled 1,310 progeny trees from 136 full‐sib families (see Table 1 for descriptive statistics) on which phenotypic measurements were taken. Most families were present on two sites, but a few of them (27) were sampled from a single site. Families were planted in selection plots of 36–48 trees and were present in only one of the tests present on any given site. The spacing of the selection plantation trials was about 2 m × 2 m with regular suppression of competing vegetation. Total tree height and diameter at breast height (DBH) were measured in 2017. Depending on the test, the age of the trees varied from 16 to 28 years (Table S1), which was taken into account in the statistical analyses. The volume of the 1,310 progeny trees measured for height and diameter at breast height (DBH) was estimated using Honer's equation (Honer, Ker, & Alemdag, 1983). All these trees were genotyped to obtain genomic profiles (see below). Two hundred and eleven (211) additional trees of the same breeding generation previously selected in the same tests were grafted and used to establish breeding gardens. These trees were also sampled and genotyped, but no phenotypic measurements were collected for them.

**TABLE 1:** Descriptive statistics of traits observed in the New Brunswick white spruce selection trials measured at all ages for the white spruce sampled and analyzed

Trait	*N*	Mean	*SD*	Min	Max	CV
HT (cm)	1,310	1,119	203.32	300	1,620	18%
DBH (mm)	1,310	155.12	32.93	38	290	21%
VOL (m^3^)	1,310	10.52	5.30	0.17	40.15	50%
VELO (Km/s)	578	3.53	0.39	2.40	4.52	11%
PICEOL (µg/mg DW)	598	7.03	4.90	0.00	26.30	70%
PUNGENOL (µg/mg DW)	598	5.87	6.05	0.00	48.00	103%
PICEIN (µg/mg DW)	598	12.07	9.56	0.00	52.70	79%

Note

The units of the traits are the original measurements. In the analyses, we transformed the Piceol and Pungenol concentration data in needles using the square root to reach the normal distribution and reduce the heterogeneity of the variance of the residuals.

Abbreviations: CV, coefficient of variation; DBH, diameter at breast height; HT, total height; Max, maximum value; Min, minimum value; PICEIN, picein concentration in needles; PICEOL, piceol concentration in needles; PUNGENOL, pungenol concentration in needles; VELO, acoustic velocity; VOL, stem volume.

Acoustic velocity was recorded as a wood quality trait for a subset of 578 progeny trees out of the 1,310 that were measured for height and DBH. Acoustic velocity is a proxy for wood stiffness or modulus of elasticity (MOE) measured at standing trees (Lenz, Auty, Achim, Beaulieu, & Mackay, 2013). We used the Hitman ST300 tool (Fibre‐Gen, New Zealand) to obtain acoustic velocity measurements of each tree. The probes were inserted approximately 4 cm into the stem and aligned vertically around breast height at a distance of approximately 70–80 cm apart. For each tree, three readings were taken on the north face of each stem and then averaged. Measurements were taken in 2018 during a period when there were no severe temperature differences that might have affected velocity measurements (Gao, Wang, Wang, & Allison, 2013).

Quantitative liquid chromatography–mass spectrometry (LC‐MS) analysis of acetophenones piceol, pungenol, and picein was conducted from needle samples for the subset of 598 progeny trees. In addition to determining the concentrations of the two acetophenone aglycones piceol and pungenol found in needles and known to be the active compounds related to spruce budworm resistance, concentrations of the constitutive glycosylated acetophenone picein were also determined as a positive control to monitor for the efficiency of acetophenone extraction from spruce needles. This glycoside appears to be inactive against the spruce budworm in white spruce (Mageroy et al., 2017). Thus, this compound was not considered in genomic selection and estimation of genetic gains. For acetophenone extraction, two‐inch branch tips were collected from the upper third of the crown in September of the same year (2018). In the field, samples were immediately put in a cooler, stored at −20°C until shipping to the Michael–Smith Laboratories at the University of British Columbia for analysis. Polar metabolites were extracted as described in the following protocol adapted from Mageroy et al. (2015) for high‐throughput analysis. Briefly, approximately 10 frozen needles were transferred to a prechilled 2‐ml tube with two 3.2 mm and one 5 mm steel balls, and ground in a mixer mill (Retsch MM 400, frequency setting 25) for 45 s. Grinding was repeated 2–6 times until needles were ground to powder as judged by visual inspection. Sample holders were chilled in liquid nitrogen between rounds of grinding to prevent the tissue from thawing. Ground needle tissue (~15 mg) was transferred with a chilled spatula to a preweighed GC vial containing 1 ml methanol with benzoic acid as internal standard (10 mg/ml). Metabolite extraction was conducted at 4°C for 24 hr in an orbital shaker. After extraction, samples were centrifuged at 250 *g* for 5 min at 4°C and the extract was transferred to a new vial for LC‐MS analysis. Tissue dry weight was determined after drying GC vials for 24 hr at 60°C in a convection oven. Extracts were stored at −80°C until LC‐MS analysis. Extraction efficiencies of acetophenones from ground needles were estimated by re‐extracting ground tissue two consecutive times with new solvent. Extraction efficiencies, determined as the ratio of the compounds in the first extract divided by the total amount of compound extracted (first plus second extract), were 86%, 95%, and 93% for picein, piceol, and pungenol, respectively.

Samples were analyzed on an Agilent 1100 LC/MSD Trap XCT Plus with an Agilent Zorbax SB‐C18 4.6 × 150 mm, 5‐micron pore size column. Solvent A was water with 0.2% (v/v) formic acid; solvent B was acetonitrile with 0.2% (v/v) formic acid. The following gradient was used: start at 5% solvent B and hold for 0.2 min; increase to 10% solvent B and hold from 0.2 to 0.5 min; increase to 15% solvent B and hold from 0.5 to 4.0 min; increase to 90% solvent B and hold from 4.0 to 7.0 min; hold at 90% solvent B from 7.0 to 8.0 min; decrease to 5% solvent B and hold from 8.0 to 8.1 min; and hold at 5% solvent B from 8.1 to 12 min. Injections of 5 µl per sample were performed with a column flow rate of 0.65 ml/min and a column temperature of 70°C. The mass spectrometer was run under electrospray ionization in negative mode, with the nebulizer pressure at 60 psi, dry gas flow rate of 12 L/min, and dry temperature of 350°C. The mass scan range was 50–400. Acetophenone peaks were extracted using the following mass ions: 343 (picein, formate adduct), 135 (piceol, parent mass), and 151 (pungenol, parent mass). Authentic standards of picein, piceol, and pungenol were used to prepare standard curves for quantitation and verify peak identities in needle extracts (Toronto Research Chemicals Inc.; Sigma‐Aldrich).

### 2.2 SNP genotyping

Previous to this study, a large registry of gene SNP markers (Pavy, Deschênes, et al., 2013) was constructed for white spruce from extensive EST resequencing and by using the reference white spruce gene catalog GCAT based on full‐length cDNAs for aligning sequences and identifying SNPs (Rigault et al., 2011). A total of 213 K high‐confidence annotated SNP markers in ~15K genes were discovered and released in the public domain (Pavy, Deschênes, et al., 2013). These markers were used to build the white spruce genotyping chip to obtain a multilocus genomic profile for each of the white spruce trees analyzed here.

From these, 5,308 SNPs representing as many different gene loci well distributed over the 12 chromosomes of white spruce (Pavy et al., 2017) and also used on previous white spruce genotyping chips (Beaulieu, Doerksen, Clément, MacKay, & Bousquet, 2014; Pavy, Gagnon, et al., 2013) were successfully manufactured to build a new Infinium iSelect genotyping array (Illumina) (Lenz, Nadeau, Azaiez, et al., 2020). Genotyping was conducted by Neogen Canada. Using replicated control samples on each sample plate, the reproducibility rate of the assay was estimated at 99.98%. After removing the nonsegregating markers and applying in‐house quality control filters on each SNP such as low rate of missing data (<15%, average of ~0.4%), a minor allele frequency (MAF) ≥0.01, an absolute value of fixation index |Fe| < 0.50, low genotyping error rate (<2%, average of 0.02%) as estimated from replicated control samples, and testing for Mendelian segregation of each SNP within full‐sib families as previously described (Lenz, Nadeau, Azaiez, et al., 2020), high‐quality genotyping data from 4,148 SNPs were retained for GS analyses. All trees from the progeny tests and the breeding gardens (total of 1,521 trees) were successfully genotyped. Five progeny trees were filtered out due to low call rate, because of insufficient good‐quality DNA. Thus, GS predictions could be made for 1,516 white spruce trees. Missing genotypes (only 0.2% of genotypes) were imputed using a k‐nearest neighbor method based on linkage disequilibrium (LD‐kNNi) with the software LinkImpute (Money et al., 2015). An accuracy of 0.79 for imputed genotypes was estimated by randomly masking 10,000 genotypes.

### 2.3 Relationship matrices

All analyses were performed in the R v.3.6.1 environment (R Core Team, 2019). A pedigree‐based relationship matrix (***A***) was first computed based on the registered pedigree information using the function “ainverse” of the R package ASReml‐R v.4.1 (Butler, Cullis, Gilmour, & Gogel, 2017). For use in genomic selection (GS) models, the realized genomic relationship matrix (***G***) was computed with the “A.mat” function of the R package rrBLUP (Endelman & Jannink, 2012) with the default options, which is equivalent to the formula described by VanRaden (2008).

### 2.4 Heritability and genotype‐by‐environment interactions

For each trait, variance components, heritability, and breeding values were estimated using the conventional pedigree‐based (ABLUP) or the genomic‐based (GBLUP) individual‐tree mixed models in ASReml‐R v.4.1:

(1) $$y=X\beta +Z_{1}a+e,$$

where ***y*** is the phenotype; ***β*** represents vectors of fixed effects including the overall mean, the site, the test within site, and the age of the trees fitted as a covariate; ***a*** is the random additive genetic effect, with $a∼N\left(0,\sigma_{a}^{2}A\right)$; and ***e*** is the residual term, with ***e*** ~ *N*(**0**, ***R***), where ***R*** is a block diagonal matrix specifying a heterogeneous error variance structure for the five sites. For the ABLUP method, the matrix ***A*** is the pedigree‐based relationship matrix, which was replaced by the realized genomic relationship matrix ***G*** for the GBLUP method ($a∼N\left(0,\sigma_{a}^{2}G\right)$). The matrices ***X*** and ***Z*_1_** are incidence matrices of their corresponding effects. The interaction of site with additive genetic effects, or genotype‐by‐environment interactions, could not be accurately estimated because the majority of families were present in only one or two sites.

The dominance effect was evaluated using the following additive–dominance model:

(2) $$y=X\beta +Z_{1}{a+Z}_{2}d+e,$$

where ***y***, ***β***, ***a***, and ***e*** are as defined in Equation 1, ***d*** is the random dominance genetic effect estimated using the family source of variation ($\sigma_{f}^{2}$) for the ABLUP model, with $d∼N\left(0,\sigma_{f}^{2}I\right)$, or using the realized dominance relationship matrix (***G*_Dom_**) calculated following Vitezica, Varona, and Legarra (2013), with $d∼N\left(0,\sigma_{d}^{2}G_{\mathrm{Dom}}\right)$ for the GBLUP model. The matrix ***I*** is an identity matrix. To test the hypothesis of greater than zero variance for additive and dominance effects (H_0_: *σ* ^2^ = 0; H_1_: *σ* ^2^ > 0), we performed a likelihood‐ratio test between the full model (Equation 1 or 2) and a reduced model without the effect to be tested.

Individual narrow‐sense heritability (${\hat{h}}_{\text{ind}}^{2}$), broad‐sense heritability (${\hat{H}}_{\text{ind}}^{2}$), and the portion of individual phenotypic variation due to dominance (${\hat{d}}_{\text{ind}}^{2}$) were estimated as:

(3) $${\hat{h}}_{\text{ind}}^{2}={\hat{\sigma }}_{a}^{2}/\left({\hat{\sigma }}_{a}^{2}+\bar{{\hat{\sigma }}_{e}^{2}}\right)\;\text{with}\;\text{model}\;\left[1\right],\;\text{or}\;{\hat{h}}_{\text{ind}}^{2}={\hat{\sigma }}_{a}^{2}/\left({\hat{\sigma }}_{a}^{2}+{\hat{\sigma }}_{d}^{2}+\bar{{\hat{\sigma }}_{e}^{2}}\right)\;\text{with}\;\text{model}\;\left[2\right],$$

(4) $${\hat{d}}_{\text{ind}}^{2}=\left(4*{\hat{\sigma }}_{f}^{2}\right)/\left({\hat{\sigma }}_{a}^{2}+{\hat{\sigma }}_{f}^{2}+\bar{{\hat{\sigma }}_{e}^{2}}\right)\;\text{for}\;\text{the}\;\text{ABLUP}\;\text{model}\;\left[2\right],$$

(5) $${\hat{d}}_{\text{ind}}^{2}={\hat{\sigma }}_{d}^{2}/\left({\hat{\sigma }}_{a}^{2}+{\hat{\sigma }}_{d}^{2}+\bar{{\hat{\sigma }}_{e}^{2}}\right)\;\text{for}\;\text{the}\;\text{GBLUP}\;\text{model}\;\left[2\right],$$

(6) $${\hat{H}}_{\text{ind}}^{2}=\left({\hat{\sigma }}_{a}^{2}+4*{\hat{\sigma }}_{f}^{2}\right)/\left({\hat{\sigma }}_{a}^{2}+{\hat{\sigma }}_{f}^{2}+\bar{{\hat{\sigma }}_{e}^{2}}\right)\;\text{for}\;\text{the}\;\text{ABLUP}\;\text{model}\;\left[2\right],$$

(7) $${\hat{H}}_{\text{ind}}^{2}=\left({\hat{\sigma }}_{a}^{2}+{\hat{\sigma }}_{d}^{2}\right)/\left({\hat{\sigma }}_{a}^{2}+{\hat{\sigma }}_{d}^{2}+\bar{{\hat{\sigma }}_{e}^{2}}\right)\;\text{for}\;\text{the}\;\text{GBLUP}\;\text{model}\;\left[2\right],$$

where $\bar{{\hat{\sigma }}_{e}^{2}}$ is the average residual error of the five sites. Standard errors of heritability estimates were obtained using the delta method (vpredict function from the ASReml‐R v.4.1 package). For each trait, estimated breeding values (EBVs from ABLUP model) or genomic estimated breeding values (GEBVs from GBLUP model) for the 1,516 trees were obtained from model [1] as the best linear unbiased predictions (BLUPs) of the random additive effect (***a***). Estimated genetic values (EGVs from ABLUP model) or genomic estimated genetic values (GEGVs from GBLUP model) of individual trees were obtained from model [2] by adding the BLUPs of the additive effect (***a***) to the BLUPs of the dominance effect (***d***).

### 2.5 Correlations between traits

To estimate phenotypic and genetic correlations between traits, bivariate models were run for all pairs of traits in ASReml‐R. The following additive‐only model was fitted:

(8) $$\left[\begin{array}{c} y_{1}\\ y_{2} \end{array}\right]=X\beta +Z_{1}a\left(t\right)+e,$$

where ***y*_1_** and ***y*_2_** are the stacked vectors of phenotypic observations for trait 1 and trait 2 respectively; ***β*** is the vector of fixed effects, including an overall mean for each trait, the site within trait, the test within site and within trait, and the age of the trees within trait; ***a***(***t***) is the random additive effect within trait, with $a\left(t\right)∼N\left(0,\;V_{A}⊗A\right)$; and ***e*** is the residual error, with $e∼N(0,\;I_{e}⊗V_{R})$. The matrix ***A*** (ABLUP) was replaced by the realized genomic relationship matrix (***G***) for the GBLUP method. The total genetic correlations (additive and dominance effects) were estimated using the following model:

(9) $$\left[\begin{array}{c} y_{1}\\ y_{2} \end{array}\right]=X\beta +Z_{1}a\left(t\right)+Z_{2}d\left(t\right)+e,$$

where ***y*_1_**, ***y*_2_**, ***β***, ***a***(***t***), and ***e*** are as defined in Equation 8, ***d***(***t***)is the random dominance genetic effect within trait estimated using the family source of variation for the ABLUP model, with $d\left(t\right)∼N\left(0,\;V_{D}⊗I_{f}\right)$, or using the realized dominance relationship matrix (***G*_Dom_**) for the GBLUP model, with $d\left(t\right)∼N\left(0,\;V_{D}⊗G_{\mathrm{Dom}}\right)$. The matrices ***V_A_***, ***V_D_***, and ***V_R_*** in Equations 8 and 9 are 2 × 2 variance–covariance matrices defined by the correlation of effects between traits (*r_a_*, *r_d_*, and *r_e_*, respectively) and unique variances for each trait (i.e., CORGH variance structure in ASReml). To facilitate convergence, we provided starting values for the variance components in ***V_A_***, ***V_D_***, and ***V_R_*** matrices that were taken from the results of the single‐trait models (Equation 1 or 2). For *r_a_*, *r_d_*, and *r_e_*, the starting value were set to 0 (i.e., no correlation), but this parameter was allowed to vary in the model. The additive genetic correlation between traits was directly given by the estimated parameter ${\hat{r}}_{a}$ from model [8], and the total genetic correlation was calculated from model [9] as:

(10) $${\hat{r}}_{g}=\frac{{\hat{r}}_{a}\sqrt{{\hat{\sigma }}_{a1}^{2}+{\hat{\sigma }}_{a2}^{2}}+{\hat{r}}_{d}\sqrt{{\hat{\sigma }}_{d1}^{2}{\hat{\sigma }}_{d2}^{2}}}{\sqrt{\left({\hat{\sigma }}_{a1}^{2}+{\hat{\sigma }}_{d1}^{2}\right)\left({\hat{\sigma }}_{a2}^{2}+{\hat{\sigma }}_{d2}^{2}\right)}}$$

where ${\hat{\sigma }}_{a1}^{2}$ and ${\hat{\sigma }}_{d1}^{2}$ are the estimated additive and dominance variance (family variance in the case of ABLUP) of trait 1 (same for trait 2), respectively. The phenotypic correlations were calculated as:

(11) $${\hat{r}}_{p}=\frac{{\hat{r}}_{a}\sqrt{{\hat{\sigma }}_{a1}^{2}+{\hat{\sigma }}_{a2}^{2}}+{\hat{r}}_{e}\sqrt{{\hat{\sigma }}_{e1}^{2}{\hat{\sigma }}_{e2}^{2}}}{\sqrt{\left({\hat{\sigma }}_{a1}^{2}+{\hat{\sigma }}_{e1}^{2}\right)\left({\hat{\sigma }}_{a2}^{2}+{\hat{\sigma }}_{e2}^{2}\right)}}\;\text{with}\;\text{model}\;\left[8\right],$$

(12) $${\hat{r}}_{p}=\frac{{\hat{r}}_{a}\sqrt{{\hat{\sigma }}_{a1}^{2}+{\hat{\sigma }}_{a2}^{2}}+{\hat{r}}_{d}\sqrt{{\hat{\sigma }}_{d1}^{2}{\hat{\sigma }}_{d2}^{2}}+{\hat{r}}_{e}\sqrt{{\hat{\sigma }}_{e1}^{2}{\hat{\sigma }}_{e2}^{2}}}{\sqrt{\left({\hat{\sigma }}_{a1}^{2}+{\hat{\sigma }}_{d1}^{2}+{\hat{\sigma }}_{e1}^{2}\right)\left({\hat{\sigma }}_{a2}^{2}+{\hat{\sigma }}_{d2}^{2}+{\hat{\sigma }}_{e2}^{2}\right)}}\;\text{with}\;\text{model}\;\left[9\right],$$

where ${\hat{\sigma }}_{a1}^{2}$, ${\hat{\sigma }}_{a2}^{2}$, ${\hat{\sigma }}_{d1}^{2}$, and ${\hat{\sigma }}_{d2}^{2}$ are defined as above, and ${\hat{\sigma }}_{e1}^{2}$ and ${\hat{\sigma }}_{e2}^{2}$ are the residual variance of trait 1 and 2, respectively. The significance of the genetic correlations was tested by performing a likelihood‐ratio test between the full model in Equation 8 or 9 and a reduced model assuming *r_a_* = 0 and *r_d_* = 0 (*i.e*., diagonal ***V_A_*** and ***V_D_*** matrices). The significance of the phenotypic correlations was tested by performing a likelihood‐ratio test between the full model in Equation 8 or 9 and a reduced model assuming no correlation between traits (i.e., fixing the parameters *r_a_* = 0, *r_d_* = 0, and *r_e_* = 0).

### 2.6 Cross‐validations

The prediction accuracy of conventional pedigree‐based (ABLUP) and genomic selection (GBLUP) models was estimated using the following procedure. For growth traits, the full set of 1,310 phenotyped individual trees was randomly split into 10 folds, each containing ~1/10th of the trees from each family. For each round of CV, nine folds (~1,179 trees) were used in model training, which was used to predict the breeding or genetic values, depending on the model used, for the remaining fold (~131 validation trees). This 10‐fold cross‐validation was repeated 10 times, for a total of 100 models for each trait. For acetophenone aglycones and acoustic velocity, respectively 598 and 578 progeny trees were successfully phenotyped, and thus, each fold used for model training contained respectively 540 and 520 trees, with the remaining phenotyped trees for these traits set aside for model validation.

The predictive ability (*PA*) of the models was evaluated as the Pearson correlation coefficient between the predicted breeding or genetic values of the validation trees and the adjusted phenotypes (y*). Adjusted phenotypes were obtained by taking the residuals (y* = ***e***) of a model that included an overall mean, the site, the test within site, and the age fixed effects as described in Equation 1. The prediction accuracy is generally defined as the correlation between the predicted and true breeding or genetic values. However, these true values are not known. Hence, the prediction accuracy (*PACC*) was estimated in three different ways: (a) first prediction accuracy was estimated from *PA* as $PACC=PA/\sqrt{{\hat{h}}_{\text{ind}}^{2}}$ for the model in Equation 1 (Dekkers, 2007; Legarra, Robert‐Granié, Manfredi, & Elsen, 2008), or $PACC=PA/\sqrt{{\hat{H}}_{\text{ind}}^{2}}$ for the model in Equation 2. To evaluate *PACC* of both ABLUP and GBLUP models, we used the ${\hat{h}}_{\text{ind}}^{2}$ estimated from the additive effects GBLUP model (Equation 3), or the ${\hat{H}}_{\text{ind}}^{2}$ estimated from the additive–dominance effects GBLUP model (Equation 7), as our best estimate of narrow‐sense and broad‐sense heritability, respectively; (b) assuming that the EBVs or EGVs obtained from the ABLUP model in Equation 1 or 2 (i.e., using 100% of phenotypes) were the true breeding values; and (c) taking the GEBVs or GEGVs obtained from the GBLUP model in Equation 1 or 2 as the true breeding values.

The GBLUP method, which considers that a quantitative trait is under the control of a large (infinite) number of loci with small genetic effects, may not be ideally suited if acetophenone aglycone traits such as piceol and pungenol are controlled by a few genes with large effects. To consider this possibility, we also tested the BayesCπ genomic selection model, which should perform better in cases of oligogenic inheritance. In the BayesCπ method, marker effects are estimated and used to predict breeding values based on tree genotypes. We fitted the same models as in Equation [1] (additive‐only model) and [2] (additive–dominance model) in the R BGLR package v.1.0.8 (Perez & de los Campos, 2014), but with ***a*** and ***d*** being the random additive and random dominance effects of markers, respectively. The incidence matrix ***Z*_1 _*_ij_*** for the additive effects ***a*** denotes genotypes for individual *i* at marker *j* coded as the number of the rare allele (e.g., 0, 1, 2). The incidence matrix ***Z*_2 _*_ij_*** for the dominance effects ***d*** denotes genotypes for individual *i* at marker *j* coded as 0 for homozygous and 1 for heterozygous genotypes (Toro & Varona, 2010). Both ***a*** and ***d*** were modeled under BayesCπ, in which only a proportion π of markers have an effect, while a proportion (1 − π) of marker effects are shrunk toward zero. This is modeled by assigning a prior for marker effects (***a*** and ***d***) that is a mixture of a point of mass at zero and a Gaussian slab (Habier, Fernando, Kizilkaya, & Garrick, 2011). The parameter π is treated as unknown and is assigned a beta prior $\pi ∼Beta\left(p_{0},\pi_{0}\right)$, with *p*_0_ > 0 and $\pi_{0}\in \left[0,1\right]$. We used the default starting parameters provided by BGLR. Similar to ABLUP and GBLUP, we fitted a heterogeneous error variance structure for the five sites in BGLR. Each model was run for 50,000 iterations and a thinning interval of 20, with the first 15,000 iterations discarded as a burn‐in. The convergence of the models was verified by running 2 MCMC chains for each trait and by using Gelman–Rubin diagnostic plots (shrink factor < 1.1) implemented in the R package coda (Plummer, Best, Cowles, & Vines, 2006). Genomic estimated breeding values were obtained by summing over the effects of all markers estimated using the additive‐only model (Equation 1), with $GEBV_{i}=\sum_{j=1}^{m}Z_{1\;\mathrm{ij}}{\hat{a}}_{j}$, where ${\hat{a}}_{j}$ is the estimated additive effect of the *j*th marker. The genomic estimated genetic values were estimated from the additive–dominance model (Equation 2) as $GEGV_{i}=\sum_{j=1}^{m}Z_{1\;\mathrm{ij}}{\hat{a}}_{j}+\sum_{j=1}^{m}Z_{2\;\mathrm{ij}}{\hat{d}}_{j}$, where ${\hat{d}}_{j}$ is the estimated dominance effect of the *j*th marker. We evaluated the *PA* and *PACC* of the BayesCπ model using the same procedure as described above. For the calculation of $PACC=PA/\sqrt{{\hat{h}}_{\text{ind}}^{2}}$ (additive model) and $PACC=PA/\sqrt{{\hat{H}}_{\text{ind}}^{2}}$ (additive–dominance model), we used the GBLUP heritability estimates so that *PACC* for ABLUP, GBLUP, and BayesCπ can be compared on equal grounds.

## 3 RESULTS

### 3.1 Narrow‐sense heritability estimates

**FIGURE 1:**
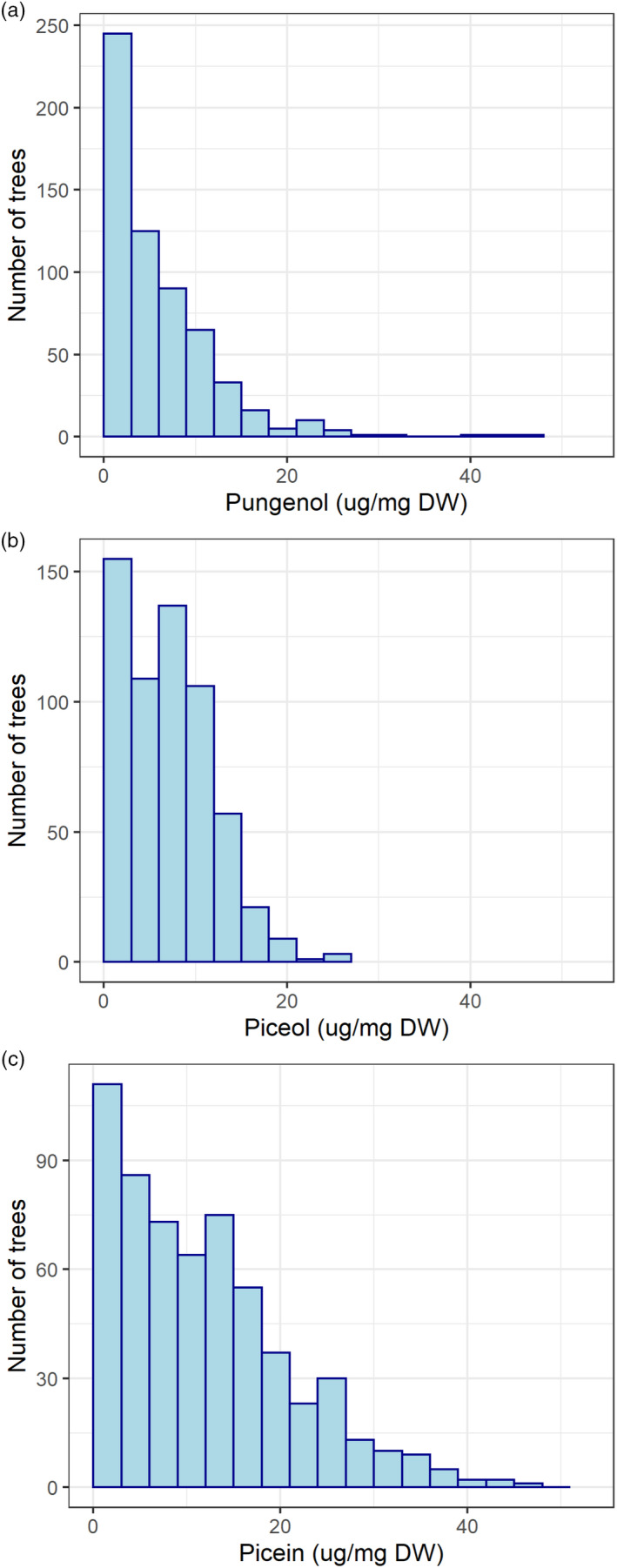
Variation of acetophenone aglycones among trees: (a) pungenol concentration in needles; (b) piceol concentration in needles; and (c) picein concentration in needles

Acetophenone aglycone data showed substantial variation among trees and families (Figure 1). They were not normally distributed and needed square root transformation in order to comply with the assumptions of normal distribution and homogeneity of the variances of modeling errors. The results of the analyses when only additive effects were considered (Equation 1) are presented in Table 2, and the additive genomic relationships among all the individuals (G‐matrix) can be seen on Figure S1. The narrow‐sense heritability estimates (${\hat{h}}_{\text{ind}}^{2}$) were significantly different from zero for all traits under both ABLUP and GBLUP models (Table 2). They were moderate to high (above 0.50 with ABLUP and 0.40 with GBLUP) for piceol, pungenol, and acoustic velocity, while those of growth traits were low to moderate (between 0.10 and 0.26). In addition, it should be noted that ${\hat{h}}_{\text{ind}}^{2}$ estimates were generally lower for GBLUP than for ABLUP. This is because the GBLUP method leads to less biased estimates of quantitative genetic parameters than ABLUP due to the use of more accurate estimates of relatedness between trees by relying on their genomic profiles (de Almeida Filho et al., 2009).

**TABLE 2:** Narrow‐sense heritability estimates for the growth, wood quality, and spruce budworm resistance traits obtained with the ABLUP and GBLUP models [1] by considering additive effects only. Standard errors are in parentheses

Trait^a^	ABLUP	GBLUP
HT	0.26 (0.06)***	0.25 (0.04)***
DBH	0.13 (0.04)***	0.13 (0.04)***
VOL	0.11 (0.04)***	0.13 (0.04)***
VELO	0.54 (0.12)***	0.41 (0.08)***
PICEOL	0.57 (0.12)***	0.43 (0.08)***
PUNGENOL	0.70 (0.13)***	0.57 (0.08)***
PICEIN	0.85 (0.13)***	0.64 (0.08)***

Note

Level of statistical significance: *p < .05; **p < .01; ***p < .001.

^a^ See Table 1 for full description of traits.

### 3.2 Additive and phenotypic correlations between traits

As anticipated, growth traits were positively correlated with each other at the phenotypic level, and phenotypic correlations between growth traits and picein, piceol, and pungenol were mostly low and negative under the GBLUP model (${\hat{r}}_{P}$ = −0.13 to 0.04) (Table S2). The additive genetic correlations between the acetophenone aglycones and growth traits were also low, negative, and generally not significant, except for a significant correlation between height and piceol under the GBLUP model (${\hat{r}}_{a}$ = −0.38 ± 0.15) (Table 3). Genetic relationships between growth traits and acoustic velocity were also low and not significant. On the other hand, piceol and pungenol concentrations were highly positively correlated (${\hat{r}}_{a}$ = 0.60 ± 0.09), whereas picein and pungenol concentrations were negatively correlated (${\hat{r}}_{a}$ = −0.65 ± 0.07) (Table 3). Altogether, the results did not show any large adverse genetic correlations between the levels of the needle aglycones piceol and pungenol on the one hand, and acoustic velocity or growth traits on the other hand.

**TABLE 3:** Estimates of the additive genetic correlation among the growth, wood quality, and spruce budworm resistance traits obtained with the ABLUP (above diagonal) and GBLUP (below diagonal) models [8] by considering additive effects only. Standard errors are in parentheses

Trait^a^	HT	DBH	VOL	VELO	PICEOL	PUNGENOL	PICEIN
HT	–	0.38 (0.17)	0.64 (0.12)**	0.14 (0.23)	−0.40 (0.19)	−0.25 (0.21)	−0.23 (0.21)
DBH	0.44 (0.14)*	–	0.77 (0.09)**	−0.28 (0.24)	−0.05 (0.26)	0.15 (0.26)	−0.41 (0.23)
VOL	0.66 (0.10)***	0.83 (0.06)***	–	−0.20 (0.26)	−0.14 (0.25)	0.09 (0.26)	−0.40 (0.24)
VELO	0.06 (0.17)	0.03 (0.21)	−0.04 (0.20)	–	−0.31 (0.17)	−0.05 (0.18)	−0.27 (0.17)
PICEOL	−0.38 (0.15)*	−0.25 (0.19)	−0.24 (0.19)	−0.20 (0.14)	–	0.59 (0.11)***	−0.03 (0.17)
PUNGENOL	−0.14 (0.16)	0.07 (0.19)	0.06 (0.19)	−0.04 (0.14)	0.60 (0.09)***	–	−0.68 (0.09)***
PICEIN	−0.11 (0.16)	−0.21 (0.19)	−0.23 (0.19)	−0.20 (0.13)	−0.03 (0.13)	−0.65 (0.07)***	–

Note

Level of statistical significance: *p < .05; **p < .01; ***p < .001.

^a^ See Table 1 for full description of traits.

### 3.3 Broad‐sense heritability estimates and genotypic correlations

ABLUP and GBLUP models that included both the additive and the dominance effects (AD) (Equation 2) were used to obtain estimates of total genetic values. Dominance genomic relationships among all the individuals (D‐matrix) are presented in Figure S2. Narrow‐sense, dominance, and broad‐sense heritability estimates can be found in Table 4. When comparing the estimates of narrow‐sense heritability obtained with the additive effects models (Table 2) to those of AD effects models (Table 4), we found a large reduction in additive genetic variance for growth traits under the AD model, which was especially apparent for the ABLUP models. For these traits, most of the genetic variance was assigned dominance variance under the AD model. For the other traits, both estimates of narrow‐sense heritability were of the same order of magnitude, especially for piceol and pungenol, for which dominance effects appeared to be low. Dominance effects were weakly significant only for height growth under the ABLUP model, and for height, DBH, volume, and acoustic velocity under the GBLUP model. AIC and BIC values obtained for each model are presented in Table S4. The AIC showed that the fit of the model was similar between the additive‐only effects models and the AD effects models, except for those traits where dominance was significant. However, the BIC was most of the time at the advantage of the additive effects model, except for height where the GBLUP AD model provided a better fit.

**TABLE 4:** Narrow‐sense, dominance, and broad‐sense heritability estimates for the growth, wood quality, and spruce budworm resistance traits obtained with the ABLUP and GBLUP models [2] by considering additive and dominance (AD) effects. Standard errors are in parentheses

Trait^a^	ABLUP			GBLUP		
	${\hat{h}}_{\text{ind}}^{2}$	${\hat{d}}_{\text{ind}}^{2}$	${\hat{H}}_{\text{ind}}^{2}$	${\hat{h}}_{\text{ind}}^{2}$	${\hat{d}}_{\text{ind}}^{2}$	${\hat{H}}_{\text{ind}}^{2}$
HT	0.01 (0.10)	0.51 (0.23)**	0.52 (0.14)***	0.18 (0.05)***	0.14 (0.05)**	0.33 (0.05)***
DBH	0.01 (0.08)	0.25 (0.17)	0.26 (0.11)***	0.09 (0.04)**	0.10 (0.06)*	0.19 (0.05)***
VOL	0.02 (0.07)	0.22 (0.16)	0.24 (0.11)***	0.09 (0.04)**	0.12 (0.06)*	0.20 (0.05)***
VELO	0.27 (0.27)	0.46 (0.50)	0.73 (0.27)***	0.30 (0.09)***	0.25 (0.10)**	0.55 (0.10)***
PICEOL	0.57 (0.23)*	0.00 (0.35)	0.57 (0.19)***	0.40 (0.09)***	0.05 (0.08)	0.45 (0.09)***
PUNGENOL	0.59 (0.27)*	0.19 (0.44)	0.78 (0.24)***	0.54 (0.09)***	0.08 (0.10)	0.62 (0.10)***
PICEIN	0.85 (0.24)*	0.00 (0.37)	0.85 (0.21)***	0.64 (0.08)***	0.00 (0.00)	0.64 (0.08)***

Note

Level of statistical significance: *p < .05; **p < .01; ***p < .001.

^a^ See Table 1 for full description of traits.

The broad‐sense heritability estimates (${\hat{H}}_{\text{ind}}^{2}$) were higher for acoustic velocity, picein, piceol, and pungenol than for the other traits, with values varying between 0.57 and 0.85 with ABLUP, and between 0.45 and 0.64 with GBLUP. The total genetic correlations (AD effects; Table 5), as well as the phenotypic correlations (Table S3), between traits were generally of the same order of magnitude and in the same direction than the additive genetic correlations and the phenotypic correlations estimated with the additive‐only effects models (Table 3 and Table S2).

**TABLE 5:** Estimates of the total genetic correlation among the growth, wood quality, and spruce budworm resistance traits obtained with the ABLUP (above diagonal) and GBLUP (below diagonal) models [9] by considering additive and dominance (AD) effects. Standard errors are in parentheses

Trait^a^	HT	DBH	VOL	VELO	PICEOL	PUNGENOL	PICEIN
HT	–	0.17 (0.33)	0.58 (0.23)**	0.14 (0.34)	−0.47 (0.24)	−0.09 (0.31)	−0.34 (0.28)
DBH	0.55 (0.11)*	–	0.73 (0.15)**	−0.08 (0.37)	−0.06 (0.37)	0.19 (0.37)	−0.48 (0.28)
VOL	0.69 (0.08)***	0.88 (0.04)***	–	−0.05 (0.36)	−0.16 (0.35)	0.15 (0.36)	−0.46 (0.25)
VELO	0.12 (0.16)	−0.15 (0.20)	−0.19 (0.18)	–	−0.34 (0.24)	−0.16 (0.25)	−0.37 (0.25)
PICEOL	−0.43 (0.15)*	−0.24 (0.19)	−0.24 (0.18)	−0.17 (0.15)	–	0.61 (0.14)***	−0.03 (0.17)
PUNGENOL	−0.14 (0.16)	0.09 (0.20)	0.09 (0.18)	−0.06 (0.14)	0.63 (0.08)***	–	−0.68 (0.11)***
PICEIN	−0.09 (0.16)	−0.18 (0.20)	−0.18 (0.18)	NA^b^	−0.03 (0.14)	−0.63 (0.08)***	–

Note

Level of statistical significance: *p < .05; **p < .01; ***p < .001.

^a^ See Table 1 for full description of traits.

^b^ The model did not converge.

### 3.4 Prediction accuracy of single‐trait selection models

#### 3.4.1 Additive effects

Results of the cross‐validation of additive‐only effects prediction models (Equation 1) for both the pedigree‐based (ABLUP) and the marker‐based (GBLUP) models are presented in Table 6. The predictive ability (*PA*) varied from about 0.40 to 0.48 for aglycones and acoustic velocity, and from about 0.11 to 0.26 for growth traits. Higher *PA* values for aglycones and acoustic velocity were anticipated given the higher heritability estimates for these traits. *PA* was of the same order of magnitude between the ABLUP and the GBLUP models, but it was slightly larger for three of the six traits (DBH, volume, and pungenol) under the GBLUP models. The first prediction accuracy (*PACC*) estimates were obtained from standardizing *PA* by the square root of the GBLUP narrow‐sense heritability estimates. Even after this standardization, *PACC* estimates were much higher for acetophenone aglycones and acoustic velocity (0.61–0.70) than for growth traits (0.31–0.52). The differences in *PACC* between ABLUP and GBLUP models were only due to differences in *PA* since we used the same heritability estimates for both types of models. Similar to *PA* values, the *PACC* estimates increased by 0.02–0.07 for three traits (DBH, volume, and pungenol) under the GBLUP models, as compared to ABLUP. *PACC* was slightly larger under ABLUP only for piceol (+0.02). The other *PACC* estimates were based on the assumptions that the true breeding values (TBVs) were obtained either from ABLUP or from GBLUP models with 100% phenotypes, and were higher than those obtained with the first method ($PA/\sqrt{{\hat{h}}_{\text{ind}}^{2}}$). The highest values were observed for both types of models when considering that the TBVs were obtained with the same models. Hence, depending on the trait, they varied from 0.70 to 0.82 for the ABLUP model with the TBVs obtained from ABLUP and from 0.81 to 0.86 for the GBLUP model with the TBVs obtained from GBLUP. Thus, we found slightly higher *PACC* for all traits with the GBLUP models using this approach to estimate *PACC*. We also tested the BayesCπ additive‐only models and found similar *PA* and *PACC* values than with GBLUP for growth traits, but slightly lower *PA* and *PACC* values for acoustic velocity, piceol, and pungenol (Table 6), indicating that the BayesCπ model assuming that the acetophenone aglycones are controlled by a few genes with large effects was not more accurate than GBLUP, which assumes a large number of genes with small effects. Moreover, the proportion π of markers having an effect was quite large for all traits, with values varying from 0.40 to 0.44 for growth traits, 0.70 for acoustic velocity, 0.56 for piceol, and 0.40 for pungenol, in support of the polygenic model.

**TABLE 6:** Predictive ability (*PA*) and prediction accuracy (*PACC*) for the growth, wood quality, and spruce budworm resistance traits obtained with the additive‐only effects models ABLUP, GBLUP, and BayesCπ (Equation 1)

Trait^a^	*PA*	Std. Err.	*PACC* ^b^	Std. Err.	*PACC* ^c^	Std. Err.	*PACC* ^d^	Std. Err.
ABLUP								
HT	0.26	0.003	0.52	0.006	0.80	0.002	0.72	0.002
DBH	0.13	0.005	0.35	0.014	0.82	0.004	0.74	0.004
VOL	0.11	0.006	0.31	0.018	0.82	0.005	0.72	0.006
VELO	0.40	0.002	0.62	0.004	0.70	0.002	0.75	0.002
PICEOL	0.46	0.002	0.70	0.000	0.71	0.002	0.77	0.002
PUNGENOL	0.46	0.002	0.61	0.003	0.72	0.002	0.75	0.002
GBLUP								
HT	0.25	0.004	0.51	0.009	0.69	0.003	0.84	0.002
DBH	0.15	0.005	0.41	0.015	0.74	0.004	0.86	0.003
VOL	0.14	0.006	0.38	0.017	0.73	0.005	0.85	0.005
VELO	0.40	0.008	0.63	0.013	0.65	0.007	0.81	0.005
PICEOL	0.44	0.007	0.67	0.010	0.66	0.006	0.82	0.004
PUNGENOL	0.48	0.006	0.63	0.009	0.68	0.005	0.81	0.004
BayesCπ								
HT	0.25	0.008	0.49	0.015	0.68	0.009	0.82	0.010
DBH	0.15	0.006	0.40	0.016	0.72	0.007	0.84	0.008
VOL	0.14	0.006	0.39	0.017	0.71	0.006	0.83	0.008
VELO	0.38	0.014	0.59	0.022	0.61	0.017	0.77	0.016
PICEOL	0.41	0.015	0.63	0.023	0.62	0.015	0.78	0.016
PUNGENOL	0.47	0.016	0.62	0.021	0.67	0.016	0.78	0.015

Abbreviations: EBV, estimated breeding value; GEBV, genomic estimated breeding value; TBV, true breeding value.

^a^ See Table 1 for full description of traits.

^b^ *PACC* = *PA*/sqrt(${\hat{h}}^{2}$). We used the ${\hat{h}}^{2}$ estimated using GBLUP for the calculation of *PACC* for ABLUP, GBLUP, and BayesCπ.

^c^ *PACC* = Corr(EBV or GEBV, TBV), where TBV = EBV obtained from the ABLUP model [1] with 100% of phenotypes

^d^ *PACC* = Corr(EBV or GEBV, TBV), where TBV = GEBV obtained from the GBLUP model [1] with 100% of phenotypes.

#### 3.4.2 Additive and dominance effects

Cross‐validation of AD effects selection models (Equation 2) were used to estimate the precision of genetic value estimates for both pedigree‐based (ABLUP) and marker‐based (GBLUP) models. A comparison of the *PA* of the additive‐only (Table 6) and the AD effects models (Table 7) indicates that they were similar, with values varying from 0.11 to 0.46, and 0.12 to 0.47, respectively, for the ABLUP models, and from 0.14 to 0.48, and 0.15 to 0.48, respectively, for the GBLUP models. However, after standardizing *PA* by the square root of the GBLUP broad‐sense heritability estimates, the prediction accuracy (*PACC*) estimates obtained with the AD effects models were slightly lower than those obtained with the additive‐only effects models, even for the traits that showed significant dominance effects (Table 4). Similar to additive‐only effects models, the lowest estimates were obtained with the method based on $PA/\sqrt{{\hat{H}}_{\text{ind}}^{2}}$ and varied from 0.27 to 0.68 for ABLUP and from 0.33 to 0.65 for GBLUP (Table 7). Slightly higher accuracies were obtained for all traits, except for piceol, under GBLUP as compared to ABLUP. The prediction accuracy based on true genetic values (TGVs) ranged from 0.60 to 0.97, depending on the method used and the how the true genetic values were estimated. When *PACC* was estimated using TGVs, we observed the same general trend as for the additive‐only effects models (Table 6). For GBLUP and using the TGVs obtained from the GBLUP model, the accuracy of the AD model was lower than that of the additive‐only effects model. However, the opposite was observed for the ABLUP model and using the TGVs obtained from the ABLUP model, with the AD model having a higher accuracy than the additive‐only effects model. The accuracies of the ABLUP AD model using TGVs from ABLUP were unexpectedly high (above 0.90 for most traits and up to 0.97) and were probably overestimated.

**TABLE 7:** Predictive ability (*PA*) and prediction accuracy (*PACC*) for the growth, wood quality, and spruce budworm resistance traits obtained with the additive–dominance models ABLUP, GBLUP, and BayesCπ (Equation 2)

Trait^a^	*PA*	Std. Err.	*PACC* ^b^	Std. Err.	*PACC* ^c^	Std. Err.	*PACC* ^d^	Std. Err.
ABLUP								
HT	0.27	0.003	0.46	0.005	0.97	0.001	0.64	0.002
DBH	0.14	0.004	0.31	0.008	0.96	0.002	0.64	0.003
VOL	0.12	0.005	0.27	0.010	0.94	0.003	0.60	0.004
VELO	0.40	0.004	0.54	0.005	0.92	0.001	0.62	0.003
PICEOL	0.46	0.002	0.68	0.003	0.80	0.001	0.75	0.002
PUNGENOL	0.47	0.003	0.60	0.004	0.84	0.002	0.72	0.002
GBLUP								
HT	0.27	0.007	0.47	0.012	0.82	0.003	0.74	0.005
DBH	0.15	0.008	0.35	0.018	0.82	0.005	0.75	0.006
VOL	0.15	0.007	0.33	0.015	0.80	0.004	0.73	0.006
VELO	0.42	0.006	0.57	0.008	0.80	0.005	0.68	0.005
PICEOL	0.44	0.008	0.65	0.011	0.71	0.003	0.79	0.005
PUNGENOL	0.48	0.006	0.61	0.008	0.75	0.004	0.77	0.005
BayesCπ								
HT	0.26	0.009	0.45	0.015	0.67	0.011	0.81	0.011
DBH	0.15	0.008	0.34	0.019	0.70	0.010	0.83	0.011
VOL	0.15	0.006	0.33	0.012	0.69	0.012	0.82	0.013
VELO	0.39	0.012	0.53	0.016	0.64	0.019	0.75	0.021
PICEOL	0.42	0.014	0.63	0.021	0.67	0.011	0.78	0.017
PUNGENOL	0.47	0.010	0.59	0.013	0.69	0.014	0.78	0.016

Abbreviations: EBV, estimated breeding value; GEBV, genomic estimated breeding value; TBV, true breeding value.

^a^ See Table 1 for full description of traits.

^b^ *PACC* = *PA*/sqrt(${\hat{H}}^{2}$). We used the ${\hat{H}}^{2}$ estimated using GBLUP for the calculation of PACC for ABLUP, GBLUP, and BayesCπ.

^c^ *PACC* = Corr(EGV or GEGV, TGV), where TGV = EGV obtained from the ABLUP model [2] with 100% of phenotypes

^d^ *PACC* = Corr(EGV or GEGV, TGV), where TGV = GEGV obtained from the GBLUP model [2] with 100% of phenotypes

Regarding the BayesCπ additive and dominance effects models, the trends found (Table 7) were similar as those above for BayesCπ additive‐only effects. We found similar or slightly lower *PA* and *PACC* values using the first method of assessing accuracy ($PA/\sqrt{{\hat{H}}_{\text{ind}}^{2}}$) under the BayesCπ model as compared to the GBLUP model. However, when the TGVs were assumed to be obtained from the GBLUP model, we surprisingly found a higher accuracy of the BayesCπ model as compared to GBLUP. These discrepancies and the lower accuracy found for GBLUP (predicted GBLUP versus TGVs GBLUP) might potentially be caused by inaccurate estimates of the dominance effects under GBLUP or that this method of estimating accuracy using TGVs is not accurate. It is likely that using the first method ($PA/\sqrt{{\hat{H}}_{\text{ind}}^{2}}$) instead of TBVs and TGVs provided more robust estimates of predictive accuracy since the TBVs and TGVs are unknown. Finally, with the BayesCπ AD model, the estimated proportion of marker having an additive effect for pungenol and piceol (π = 0.41 and 0.55, respectively) or dominance effect (π = 0.37 and 0.53, respectively) was in the same range as for growth traits (additive: π = 0.36–0.43; dominance: π = 0.38–0.44), indicating a similar gene control implicating a large number of genes for all traits.

### 3.5 Genetic gain expectations from genomic selection

We estimated the genetic gains that could be expected from the selection of the top 5% trees for acetophenone aglycones based on either their GEBVs for the establishment of seed orchards, or GEGVs in the context of multiclonal/multivarietal forestry (Park, Beaulieu, & Bousquet, 2016). To do so, and given that all 1516 trees of the multisite selection population were previously genotyped, we inferred breeding and genetic values for the 916 unphenotyped candidate trees for acoustic velocity and acetophenone aglycones by using GS models built from the 598 trees that were both genotyped and phenotyped, thus allowing selection to be applied to the whole selection population. Thus, the 5% selection intensity corresponded to the selection of the top 76 trees among the 1,516 trees for which genomic profiles were obtained. The gains expected from the selection of each trait considered individually are presented on the diagonal of both subtables (Table 8). It can be seen that the gains are slightly higher when the selection is based on GEGVs as compared to GEBVs. Overall, selection at this intensity for aglycones would generate large gains (~37% for piceol and ~45%–47% for pungenol) and would generally not translate into lost in growth traits (maximum loss of 0.4 to 0.6% in volume with selection made for pungenol concentration). Loss in wood stiffness, as measured by acoustic velocity, would be slightly higher, but still not above 1.1%. The 76 progeny trees selected for superior piceol concentration belonged to 21 full‐sib families, whereas those selected for high pungenol concentration belonged to 27 full‐sib families out of the 136 families tested. However, selection for higher height growth would generate losses of up to 10%–12% and 4.8%–5.4% in piceol and pungenol concentrations, respectively. A selection for volume resulted in smaller losses in piceol and pungenol. Independent culling for the two aglycones would generate 36% and 27% gains in piceol and pungenol, respectively, when the trees are first selected for highest piceol concentration in needles and those having negative breeding values for pungenol concentration being culled. If trees were first selected for pungenol concentration in needles and then culled for piceol, the respective gains would be 27% and 42% for piceol and pungenol, respectively. The gains obtained using a selection index giving the same weight (*w* = 0.5) to both aglycones would be 32% and 41% for piceol and pungenol concentration, respectively, and the selected trees would represent 26 full‐sib families. However, following such a selection index scheme, no progress would be made for height or wood stiffness (loss of 0.03% in height and loss of 0.5% in wood acoustic velocity).

**TABLE 8:** Genetic gains (%) expected from the selection of the top 5% trees using GBLUP breeding values and genetic values. The gains expected from the selection of each trait considered individually are presented on the diagonal of both subtables. The gains expected from selecting the top 5% trees for each trait separately are indicated in rows, and the correlated genetic gains on other traits are indicated in columns. The gains are presented as a percentage of the phenotypic mean of the population. Negative numbers indicate a loss in trait values

Trait^a^	HT	DBH	VOL	VELO	PICEOL	PUNGENOL
Based on genomic estimated breeding values						
HT	7.6	3.6	10.6	2.0	−12.1	−4.8
DBH	4.8	6.6	10.6	0.4	−6.5	−2.2
VOL	5.1	6.2	17.8	−0.1	−9.7	−0.4
VELO	1.0	−0.4	−0.4	8.7	−12.1	−7.0
PICEOL	0.1	0.0	0.8	−1.1	36.6	24.8
PUNGENOL	−0.3	−0.3	−0.6	−0.5	21.2	45.4
Based on genomic estimated genetic values						
HT	8.8	4.2	13.2	1.4	−10.1	−5.4
DBH	5.5	8.0	21.4	−0.2	−5.1	−2.0
VOL	6.3	7.7	22.7	0.0	−6.6	−2.0
VELO	1.4	−0.7	−0.7	10.9	−6.8	−4.0
PICEOL	0.4	0.3	1.6	−1.1	37.3	25.6
PUNGENOL	0.1	−0.3	−0.4	0.1	20.3	47.3

^a^ See Table 1 for full description of traits.

**FIGURE 2:**
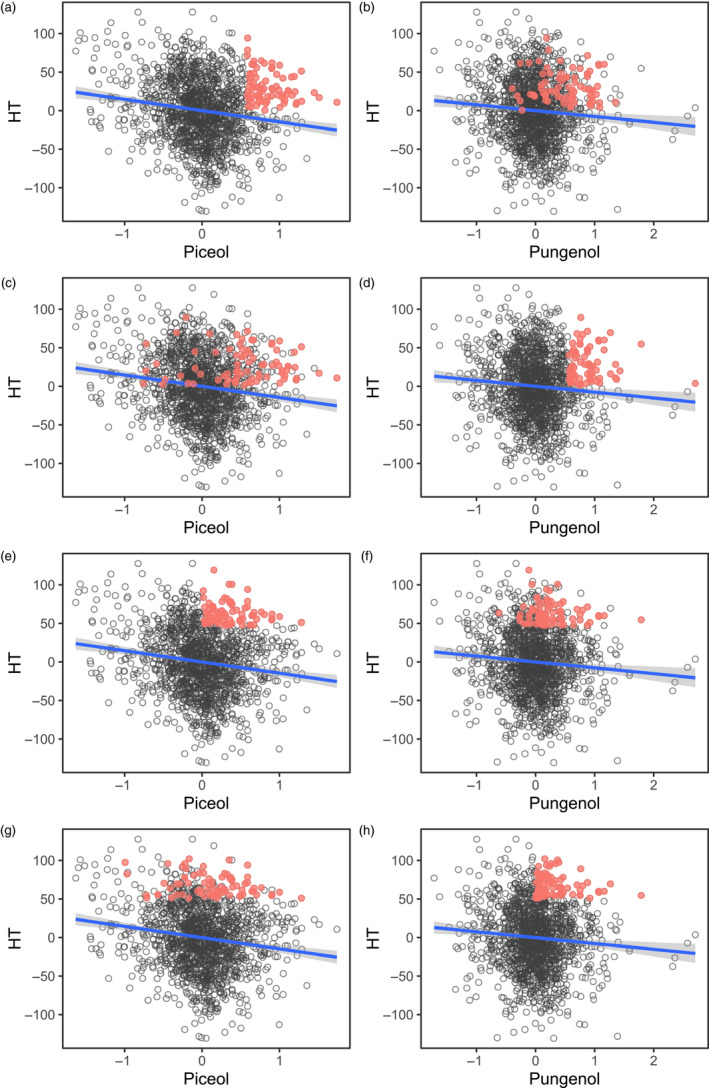
Relationships between breeding values for piceol, pungenol, and height are illustrated. Selected correlation breakers are highlighted in red for different scenarios: (a, b) show the top 5% trees for piceol and breeding values for height ≥0; (c, d) show the top 5% trees for pungenol and breeding values for height ≥0; (e, f) show the top 5% trees for height and breeding values for piceol ≥0; (g, h) show the top 5% trees for height and breeding values for pungenol ≥0

Finally, a set of correlation breakers could be identified, with improvement in acetophenone aglycones and other traits at the same time. For instance, the selection of 5% of the trees with the highest breeding values for piceol concentration in needles and positive breeding values for tree height would generate genetic gains of 32% and 2.8% for piceol concentration and tree height, respectively (Figure 2a,b; 76 trees from 22 full‐sib families). At the same time, there would be a gain of 21% for pungenol concentration, but a loss of about 1% in wood stiffness. Similarly, the selection of correlation breakers for pungenol concentration and height would generate 35% and 2.5% of genetic gains for pungenol concentration and tree height, respectively (Figure 2c,d; 76 trees from 34 full‐sib families). A gain of 20% in piceol concentration and a loss of 0.7% in wood stiffness would also be expected. If the emphasis was first put on height and culled for piceol afterward (positive breeding values), the expected gains would be 5.7%, 13%, 11%, and −0.5% for height, piceol, pungenol, and wood stiffness, respectively (Figure 2e,f; 76 trees from 33 full‐sib families). With height and pungenol concentration as the primary and secondary selected traits, respectively, the expected gains would in the same order, 6.1%, 5%, 14%, and 0.5% (Figure 2g,h; 76 trees from 33 full‐sib families). Expected genetic gains or losses, depending on the targeted traits, were of the same order of magnitude when selecting trees based on the highest genetic values instead of the highest breeding values.

## 4 DISCUSSION

The most recent spruce budworm outbreaks in the Canadian boreal forest (1950–1993) affected close to 85 million hectares, killing about half of the host trees and leading to a commercial volume loss of 3–68 m^3^/ha dependent on the intensity of the outbreak (Gray & MacKinnon, 2006). Breeding for SBW resistance could help avoid economic loss in forest plantations in the future. The acetophenone aglycones piceol and pungenol were found to play a major role in SBW resistance in white spruce, and underlying resistance mechanism was discovered in recent years as reviewed in Parent et al. (2020). Although aglycone concentrations in leaf material were shown to be under significant genetic control (Méndez‐Espinoza et al., 2018), it was unclear whether accelerated breeding through GS would be possible and would lead to meaningful increment of leaf aglycones in breeding populations. Here, we present a proof of concept for using genomic selection in the context of improving resistance against insect defoliation in a conifer and by focusing on needle compounds shown to be directly related to insect resistance. We show that the moderate‐to‐high heritability and high accuracy of GS models led to large expected genetic gains in the concentrations of piceol and pungenol in white spruce needles.

### 4.1 Strong genetic control of SBW resistance traits makes them candidate for genomic selection

The narrow‐sense heritability estimates obtained using the additive‐only effects models for acetophenone aglycones in the current study are in line with those reported by Méndez‐Espinoza et al. (2018) for 9‐year‐old tests established in Quebec and regrouping 33 full‐sib families. These authors found ${\hat{h}}_{i}^{2}$ = 0.50 ± 0.07 for piceol concentration in needles, and ${\hat{h}}_{i}^{2}$ = 0.55 ± 0.07 for pungenol concentration. For picein concentration, a constitutive glycosylated acetophenone used herein as a control, Méndez‐Espinoza et al. (2018) reported a marginally higher narrow‐sense heritability estimate (${\hat{h}}_{i}^{2}$ = 0.60 ± 0.06). In the present study, we found higher narrow‐sense heritability estimates for piceol, pungenol, and picein using ABLUP, but comparable and more realistic estimates using GBLUP. These results confirm that the genetic control of piceol and pungenol is moderate to strong in white spruce and that higher levels of accumulation of these compounds in needles could be achieved through breeding and selection.

Our results are also in line with those previously reported for other quantitative traits. Hence, Li et al. (1993) estimated narrow‐sense heritability of tree height at age 8 years for open‐pollinated families tested on three sites in southern Quebec. They found estimates varying from 0.10 to 0.17. Those reported by Beaulieu, Doerksen, MacKay, et al. (2014) for 22‐year‐old white spruces were similar to ours (${\hat{h}}_{i}^{2}$ = 0.25), whereas estimates obtained for 15‐year‐old full‐sib progeny trees were slightly higher (${\hat{h}}_{i}^{2}$ = 0.33) (Lenz et al., 2013). Globally, all of these estimates are of the same magnitude showing that the genetic control of tree height in white spruce is generally low to moderate. For acoustic velocity, Lenz et al. (2013) reported narrow‐sense heritability estimates of 0.38 in white spruce, which is slightly lower than estimates obtained in the current study. Lenz, Nadeau, Azaiez, et al. (2020) analyzed acoustic velocity data collected in both full‐sib and polycross white spruce progeny tests. Similar narrow‐sense estimates of 0.41 and 0.35 were found for the full‐sib and the polycross tests, respectively. Thus, there is agreement between these studies that acoustic velocity is under moderate genetic control in white spruce and, on average, higher than that for growth traits but lower than that for acetophenone aglycones.

It appears that the ABLUP and GBLUP models did not succeed to separate well the additive and the dominance genetic effects, which was especially apparent for the ABLUP AD models. Hence, under the ABLUP AD models, the portion of phenotypic variation due to additive genetic effects (i.e., the narrow‐sense heritability) dropped to near zero and all of the genetic variance was assigned to dominance for growth traits, indicating that additive and dominance effects were confounded. Using ABLUP, dominance was only significant for height and the standard errors of dominance estimates were large for all traits. In contrast, with GBLUP significant dominance was found for the three growth traits and acoustic velocity, with much smaller standard errors, which is in line with results reported by Muñoz et al. (2014) for tree height in loblolly pine (*Pinus taeda* L.) using a clonal trial made of 951 individuals belonging to 61 full‐sib families. These authors showed that, compared with using pedigree information, use of the realized genomic relationships in GBLUP models resulted in a substantially more precise separation of additive and nonadditive components of genetic variance. The experimental design available in the present study was not designed to obtain accurate estimates of dominance variance since each family was present in only one test (one family plot) per site in our sampled dataset, and some tests contained a small number of families. Thus, it is likely that the dominance effects of specific crosses were in part confounded with the test effects and the within‐test microenvironment differences between family plot effects.

Few studies have reported broad‐sense heritability estimates for traits of interest in white spruce. However, Méndez‐Espinoza et al. (2018) presented such estimates for piceol, pungenol, and picein concentrations from needles collected in two clonal tests in Quebec and three clonal tests in New Brunswick. The 21 clones tested in Quebec were 6 years old, whereas the 50 clones tested in New Brunswick were 14 years old. For the Quebec tests, these authors reported estimates that were of the same order of magnitude than those found in the present study (piceol, ${\hat{H}}_{i}^{2}$ = 0.66 ± 0.07; pungenol, ${\hat{H}}_{i}^{2}$ = 0.60 ± 0.08; picein, ${\hat{H}}_{i}^{2}$ = 0.77 ± 0.05). However, for older ramets in New Brunswick, their estimates were much lower (piceol, ${\hat{H}}_{i}^{2}$ = 0.29 ± 0.07; pungenol, ${\hat{H}}_{i}^{2}$ = 0.37 ± 0.06; picein, ${\hat{H}}_{i}^{2}$ = 0.23 ± 0.06), but nevertheless showing a moderate genotypic control for these traits. Unfortunately, these authors did not estimate dominance effects. Thus, further studies will be required to verify whether the low dominance effects observed in the present study are only typical of the breeding population analyzed herein. However, we consider that the results of the present study and those of previous studies support the idea that the genetic control of the acetophenone aglycones in white spruce is moderate to high and that substantial genetic gains can be expected from the selection of superior genotypes and their vegetative propagation through somatic embryogenesis or cuttings or a combination of both (Park et al., 2016).

Broad‐sense heritability was also estimated for white spruce tree height by Park, Weng, and Mansfield (2012). They reported estimates of ${\hat{H}}_{i}^{2}$ = 0.35 for tree height at age 14 using 340 clones from a population of 75 full‐sib‐families established in three locations in New Brunswick. Their estimate is in line with that obtained in the present study. Wahid et al. (2012) also reported heritability estimates for white spruce tree height using data collected in two clonal tests established in Quebec using 52 clones from 14 full‐sib families. Their estimates were slightly lower than ours with values of 0.10 and 0.26, which might be partly due to ontogenic effects in younger material. More studies would be needed to confirm this hypothesis.

#### 4.1.1 Weak evidence of a trade‐off between growth and acetophenone aglycones traits

In the present study, at both the additive genetic and the phenotypic levels, we found highly significant positive correlations between piceol and pungenol concentrations in needles and negative correlations between the concentration of picein and that of pungenol. Similar relationships between piceol and pungenol concentrations were also found at both the additive genetic and phenotypic levels (${\hat{r}}_{a}$, ${\hat{r}}_{p}$ ~ 0.80 ± 0.05), by Méndez‐Espinoza et al. (2018). However, they did not report any significant negative relationship between the acetophenone glucoside picein and pungenol concentrations in needles, contrary to us. This significant negative relationships are also in contradiction with Mageroy et al. (2015) who reported that high levels of picein were not related to the accumulation of acetophenone aglycones. This discrepancy could be due to various reasons such as differences in the provenance of the population assessed, differences in age of the material sampled (47 years versus 16–28 years in the present study), or samples having not been collected at the same time in both studies (summer versus autumn). The total genetic correlation coefficients (additive–dominance models) between the acetophenone aglycones estimated in the present study are of the same order of magnitude than those previously reported in Méndez‐Espinoza et al. (2018). However, their total genetic correlation coefficients were slightly smaller than their additive genetic correlation coefficients, contrary to the trend observed in the present study using either the ABLUP or the GBLUP models.

We also found that the acetophenone aglycone concentrations in needles were either not correlated or marginally negatively correlated with the other traits. This is also in some contrast with those reported previously by Méndez‐Espinoza et al. (2018). These authors reported highly significant positive phenotypic and genetic correlations between height and the concentration of the two acetophenone aglycones piceol and pungenol for breeding material from Québec. Although it is difficult at the present time to find the reason for such differences, the genetic background and the age of the material tested in the two studies differed: The material from Méndez‐Espinoza et al. (2018) came from southern Québec and Ontario, regions that experienced weaker or rare SBW outbreaks in the past. Those populations generally show lower concentrations of aglycones (Parent et al., 2017) than those from New Brunswick considered in our study. In addition, the material used in the present study (16–28 years old) was much older than the rather juvenile material from Méndez‐Espinoza et al. (2018) and the relationships between growth and concentrations of acetophenone aglycones in needles may change with developmental age. Different dates of needle collection (September in the current study versus July for the Méndez‐Espinoza et al., 2018 study) and laboratory practices might have influenced the results obtained (average piceol concentration: 9.60 ± 0.71 µg/mg DW versus 7.03 ± 4.90 µg/mg DW; average pungenol concentration: 17.40 ± 1.17 µg/mg DW versus 5.87 ± 6.05 µg/mg DW, respectively, in the Méndez‐Espinoza et al., 2018 study and the current study).

The general lack of trade‐off between growth and acetophenone aglycones (except for a weak negative genetic correlation between height and piceol concentration) observed from previous studies, at the phenotypic level by Lamara et al. (2018) and at both the phenotypic and genetic levels by Méndez‐Espinoza et al. (2018), was confirmed in this dataset. However, this topic needs further investigation across the sympatric area of SBW and white spruce in order to better understand the ecological and evolutionary implications of the resistance mechanism and coevolution.

#### 4.1.2 High predictive accuracy of GS models allows genomic‐assisted selections

##### Additive genetic effects

Because the true breeding values of the tested material are unknown, one preferred method to estimate predictive accuracy is to calculate the Pearson correlation coefficient between the predicted breeding values and the adjusted phenotypes, and then standardize by the square root of heritability (Lenz, Nadeau, Azaiez, et al., 2020; Lenz, Nadeau, Mottet, et al., 2020). Using this approach, we found moderate‐to‐high predictive accuracies for both ABLUP and GBLUP models, but GBLUP slightly outperformed ABLUP for DBH, volume, and pungenol concentration. Most importantly, our results indicate that genomic selection was highly precise for aglycones as we found the highest accuracies for piceol and pungenol, and for a variety of sites given that the five different test sites were simultaneously considered in the model. This increase in predictive accuracy is likely due to low genotype‐by‐environment interactions across sites for these traits as reported previously (Méndez‐Espinoza et al., 2018). Our dataset did not allow obtaining accurate estimates of such interactions and to separate them from the average across‐site effects given the unbalanced representation of families across test sites.

In this study, estimates of predictive accuracies based on “true breeding values” (TBV) obtained from the models with 100% of phenotypes were also estimated (Table 6) because this approach is very common in the literature (e.g., Beaulieu, Doerksen, Clément, et al., 2014; Chen et al., 2018; Lenz et al., 2017). We found higher predictive accuracy values using this approach, by about 1%–124% depending on the trait, especially when the same model was used to estimate reference true breeding values (i.e., ABLUP predicted versus ABLUP 100% phenotypes, or GBLUP predicted versus GBLUP 100% phenotypes). So far, such approaches based on TBVs tended to overestimate predictive accuracy, and thus, we did not put emphasis on these results.

The genetic control of plant defense sometimes conforms to an oligogenic model, especially in the case of resistance to pathogens (Walters, 2011). To test this hypothesis for acetophenone aglycones involved in herbivory defense against SBW, we used the BayesCπ approach to verify whether it would allow identifying a small number of markers with large effects conferring higher prediction accuracies than that of the GBLUP model, which considers that a quantitative trait is under the control of a large number of loci with small effects. With BayesCπ, the proportion of markers that had significant effects on aglycone additive‐only genetic variance was large (40%–56%) and in the same range than that of the other traits. Moreover, the prediction accuracy of the BayesCπ models was slightly lower than that of the GBLUP models, not higher (Table 6). Taken together, these two trends do not support an oligogenic model of gene effects for piceol and pungenol. This is not unexpected given the skewed distributions of metabolite values (Figure 1), which did not reflect a bimodal or oligomodal distribution, but a more continuous distribution indicative of multiple gene effects. The boxplot of marker effects obtained with BayesCπ shows only one marker having a marginally larger effect, for pungenol than the other SNP markers (Figure S3a). But it was still accounting for a small fraction of the total genomic estimated breeding values of the trees, and the relationship of marker effects between piceol and pungenol followed the general polygenic pattern between both traits (Figure S3b). Given that marker effects estimated with genomic selection methods are not independent and that trees were related in this advanced‐breeding population, other materials and approaches such as association and QTL mapping would help further characterize the genomic architecture of these traits. Indeed, a previous association study for piceol and pungenol on white spruce identified a large number of candidate genes with likely implications in the genetic control of these metabolites (Lamara et al., 2018), in accord with the trends observed here.

##### Additive–dominance genetic effects

In the present study, we did not find marked differences in predictive ability between the additive‐only and additive–dominance effects models, and even slightly lower predictive accuracies for the last ones. Such lack of difference in the results between these two types of models has already been noted especially for fusiform rust resistance (oligogenic trait) in a structured breeding population of loblolly pine (*Pinus taeda* L.). Indeed, de Almeida Filho et al. (2016) showed that both models were largely similar in predictive ability and that the inclusion of dominance effects in the model improved only slightly the predictions for tree height, a polygenic trait. Simulating six traits with different genetic architectures (polygenic and oligogenic), and levels of dominance effects, these authors also showed that AD models improved markedly predictive accuracies only when traits were affected by larger dominance effects (*d* ^2^ > 0.2). Bouvet, Makouanzi, Cros, and Vigneron (2016) reported similar finding for 32‐month height of *Eucalyptus urophylla* x *E. grandis* hybrids clonally replicated on three sites. Absence of improvement was also observed in other organisms, such as dairy cattle, for milk production (Ertl et al., 2014).

In this study, significant dominance effects, though still rather low (${\hat{d}}_{\text{ind}}^{2}$ = 0.10–0.25 using GBLUP), were detected for growth traits and acoustic velocity under the GBLUP model. However, dominance effects were not important in white spruce for aglycones related to resistance to spruce budworm, similar to what was found for resistance to fusiform rust in loblolly pine (de Almeida Filho et al., 2016). For tree height in white spruce, Weng, Park, Krasowski, Tosh, and Adams (2008) reported that the percentage of the phenotypic variance due to dominance varied between 10% and 15% between age 5 and 14 years. These results are in line with those of the large majority of studies made on a variety of growth and wood traits in pines, showing a net excess of additive over dominance variance (see table A1 in Bouvet, Saya, & Vigneron, 2009). Thus, the dominance effects in white spruce might not be strong enough to realize any advantage for AD over additive‐only effects models in terms of prediction accuracy. Indeed, de Almeida Filho et al. (2016) indicated that it is only once the percentage of phenotypic variance explained by the dominance effects reaches 20% that the AD models provide more accurate predictions. Furthermore, dominance effects may not well be estimated due to partial confounding with the test and family plot effects in this study.

Using the BayesCπ additive–dominance model, we found similar or slightly lower predictive accuracies than the GBLUP AD model for acetophenone aglycones, and the proportion of markers with nonzero effect (37%–55%) again did not support a hypothesis of oligogenic control of these traits (Figure S4a,b).

Overall, we conclude that the additive GBLUP model was sufficient to obtain highly accurate predictions of the concentrations of piceol and pungenol in needles using only a small subset (598 trees out of 1,310) of the breeding population. These very encouraging results should allow for significant cost savings by reducing phenotyping costs for such metabolites implicated in pest resistance, which are cumbersome and expensive to assess.

### 4.2 Important genetic gain for acetophenone aglycones would increase SBW resistance in future plantations

From this dataset, important genetic gains of more than 35 and 45% for piceol and pungenol concentrations in needles, respectively, were expected when low‐intensity selection (top 5%) was based on the respective traits. Besides genetic control, those high percentages are related to the skewed trait distribution of needle concentrations of piceol and pungenol in the population and the fact that less than a fifth of individuals carried meaningful concentrations of those molecules (Figure 1). The effects of piceol and pungenol on larvae survival and development using artificial diets were previously reported (Delvas, Bauce, Labbé, Ollevier, & Bélanger, 2011). Our population mean values for piceol and pungenol concentrations in needles are in the same range as their reference dose (2 × piceol + 2 × pungenol), which lead to 50% less survival of larvae, significant lower purple mass, and a longer development time compared with the control of no piceol and pungenol in the artificial diets. Hence, selecting individuals with strong breeding or genetic values for concentrations in piceol and pungenol should increase significantly SBW resistance in the breeding material. Although SBW would likely feed on more resistant trees, food consumption of larvae will be lower due to antidigestive or toxic effects of the more resistant foliage (Despland et al., 2011), and hence lead to less damage. In addition, bioassays may underestimate the effect of piceol and pungenol as their maximum concentrations in most testing designs are significantly lower than the reported levels in resistant trees (Delvas et al., 2011; Parent et al., 2020). Before budburst, SBW feeds on foliage from the previous year. Furthermore, as the high levels of acetophenone aglycones are usually preserved in the needles of the previous year in resistant phenotypes, it negatively affects early larvae development and survival (Parent et al., 2017). This is in contrast with insects used in experimental rearing trials that initially grow up under rather perfect controlled conditions.

In multitrait selection scenarios, loss of needle concentrations in one or both of piceol and pungenol should be avoided in order to keep trees with adequate levels of acetophenone aglycones leading to meaningful resistance in the breeding population. In this study, we were able to identify correlation breakers, that is, progeny trees that combined good growth and high levels of acetophenone aglycones leading to genetic gain for both categories of traits. Overall, a balanced gain in concentrations of both acetophenone aglycones piceol and pungenol, as well as moderate gain in growth, should be the ideal compromise scenario to look for when breeding for improved SBW resistance in white spruce. Additional field testing would be advisable to help determine the appropriate weights that should be given to each trait and to evaluate the effective gains obtained from selecting more resistant white spruce trees to SBW attacks in terms of reduced defoliation and higher growth in plantation site conditions.

## 5 CONCLUDING REMARKS

We have argued that with climate change and its potential effects on pest distributions and host–pest relationships, pest resistance should be of high priority in tree breeding programs and that the existing genetic variation for resistance traits and relationships with productivity traits should be better understood for their consideration in multitrait breeding. In this study, we evaluated the prospects for genetic improvement of the levels of acetophenone aglycones in white spruce needles, which have been shown to be tightly linked to resistance to spruce budworm. We showed that aglycones were under moderate‐to‐strong genetic control with null or marginally negative genetic correlations with other traits. The prediction accuracy of GS models for these metabolites was also high and comparable with that of pedigree‐based models. These are very encouraging result for their genetic improvement with regard to enhancing white spruce resistance to spruce budworm at a very early stage and without the need to test and phenotype all candidates. GS can thus be deployed early to conduct such selection or to infer breeding and genetic values of unphenotyped material, thus saving time and costs. Finally, we showed that various selection strategies involving GS can be used to simultaneously improve productivity and adaptation traits such as pest resistance, which are essential to face climate change pressures and help maintain health and productivity of forest plantations.

## CONFLICT OF INTEREST

None declared.

## Supporting information

Appendix S1

Appendix S2

Appendix S3

Supplementary Material

## Data Availability

In order to comply with Intellectual Property Policies (IPP) of participating governmental institutions in this work, the supporting phenotyping data are not deposited into a public domain database. The original data are stored in the institutions’ databases and may be shared upon request to the corresponding author according to our IPP. The SNP genotyping data are provided as Supporting Information. The SNP genotyping dataset will be deposited into the public domain once the manuscript is accepted.
