# Supplementary material for: Genomic selection for resistance to spruce budworm in white spruce and relationships with growth and wood quality traits
Source: Evol Appl. 2020 Aug 11;13(10):2704–22. doi: 10.1111/eva.13076 (PMC7691460; doi:10.1111/eva.13076)
Supplement: Supplementary file 4 — Supplementary Material [file EVA-13-2704-s004.docx]

**Supplementary material**

Table S1. Identification of the white spruce tests and the age of the trees in each test.

| Test site in  New Brunswick, Canada | Test number | Number of trees | Age of trees |  | Latitude (^o^, ‘ N) | Longitude  (^o^, ‘ W) |
| --- | --- | --- | --- | --- | --- | --- |
| Sussex | 97 | 8 | 28 |  | 45 44 | 65 28 |
| Black Brook | 102 | 80 | 28 |  | 46 27 | 67 28 |
| Deersdale | 104 | 8 | 28 |  | 46 31 | 67 00 |
| Sussex | 105 | 19 | 27 |  | 45 44 | 65 28 |
| Deersdale | 106 | 8 | 27 |  | 46 31 | 67 00 |
| Sussex | 128 | 3 | 26 |  | 45 44 | 65 28 |
| Black Brook | 133 | 8 | 26 |  | 46 27 | 67 28 |
| Black Brook | 135 | 40 | 26 |  | 46 27 | 67 28 |
| Black Brook | 156 | 65 | 25 |  | 46 27 | 67 28 |
| Sussex | 161 | 28 | 24 |  | 45 44 | 65 28 |
| Deersdale | 162 | 9 | 24 |  | 46 31 | 67 00 |
| Black Brook | 163 | 10 | 24 |  | 46 27 | 67 28 |
| Black Brook | 165 | 140 | 24 |  | 46 27 | 67 28 |
| Black Brook | 205 | 60 | 22 |  | 46 27 | 67 28 |
| Sussex | 206 | 20 | 22 |  | 45 44 | 65 28 |
| Black Brook | 217 | 80 | 21 |  | 46 27 | 67 28 |
| Black Brook | 229 | 40 | 20 |  | 46 27 | 67 28 |
| Black Brook | 243 | 25 | 18 |  | 46 27 | 67 28 |
| Deersdale | 264 | 20 | 16 |  | 46 31 | 67 00 |
| Dunbar | D2 | 30 | 16 |  | 46 08 | 66 36 |
| Dunbar | D2 | 25 | 18 |  | 46 08 | 66 36 |
| Dunbar | D2 | 50 | 20 |  | 46 08 | 66 36 |
| Dunbar | D1 | 125 | 21 |  | 46 08 | 66 36 |
| Dunbar | D1 | 75 | 22 |  | 46 08 | 66 36 |
| Dunbar | D1 | 55 | 24 |  | 46 08 | 66 36 |
| Dunbar | D1 | 74 | 25 |  | 46 08 | 66 36 |
| Dunbar | D1 | 100 | 26 |  | 46 08 | 66 36 |
| Wheelers Cove | WC | 105 | 24 |  | 45 52 | 66 57 |

Table S2. Estimates of the phenotypic correlations among the growth, wood quality, and spruce budworm resistance traits obtained with the ABLUP (above diagonal) and GBLUP (below diagonal) models [8] considering additive effects only. Standard errors are in parentheses.

| Trait^†^ | HT | DBH | VOL | VELO | PICEOL | PUNGENOL | PICEIN |
| --- | --- | --- | --- | --- | --- | --- | --- |
| HT | - | 0.66 (0.02)*** | 0.73 (0.01)*** | 0.01 (0.06) | -0.15 (0.05)* | -0.15 (0.05)** | 0.00 (0.06) |
| DBH | 0.66 (0.02)*** | - | 0.94 (0.00)*** | -0.17 (0.05)*** | -0.03 (0.05) | -0.07 (0.05) | 0.01 (0.05) |
| VOL | 0.73 (0.01)*** | 0.95 (0.00)*** | - | -0.13 (0.04)** | -0.04 (0.04) | -0.06 (0.05) | 0.00 (0.05) |
| VELO | -0.03 (0.05) | -0.15 (0.04)*** | -0.12 (0.04)*** | - | -0.11 (0.06) | -0.03 (0.07) | -0.11 (0.07) |
| PICEOL | -0.13 (0.05)* | -0.05 (0.04) | -0.04 (0.04) | -0.06 (0.05) | - | 0.61 (0.04)*** | -0.14 (0.07)*** |
| PUNGENOL | -0.13 (0.05)** | -0.08 (0.04)* | -0.06 (0.04) | -0.03 (0.05) | 0.61 (0.03)*** | - | -0.63 (0.04)*** |
| PICEIN | 0.04 (0.05) | 0.05 (0.04) | 0.02 (0.04) | -0.08 (0.05) | -0.16 (0.05)*** | -0.63 (0.03)*** | - |

^†^ See Table 1 for full description of traits.

Level of statistical significance: **P* < 0.05; ***P* < 0.01; ****P* < 0.001.

Table S3. Estimates of the phenotypic correlations among the growth, wood quality, and spruce budworm resistance traits obtained with the ABLUP (above diagonal) and GBLUP (below diagonal) models [9] considering additive and dominance (AD) effects. Standard errors are in parentheses.

| Trait^†^ | HT | DBH | VOL | VELO | PICEOL | PUNGENOL | PICEIN |
| --- | --- | --- | --- | --- | --- | --- | --- |
| HT | - | 0.66 (0.02) *** | 0.73 (0.01) *** | -0.01 (0.05) | -0.13 (0.05) | -0.15 (0.05) *** | 0.00 (0.06) |
| DBH | 0.66 (0.02)*** | - | 0.94 (0.00) *** | -0.17 (0.05) *** | -0.03 (0.05) | -0.07 (0.05) | 0.01 (0.05) |
| VOL | 0.73 (0.01)*** | 0.95 (0.00)*** | - | -0.13 (0.04) ** | -0.04 (0.04) | -0.06 (0.04) | 0.00 (0.04) |
| VELO | -0.02 (0.05) | -0.14 (0.04)*** | -0.12 (0.04)** | - | -0.11 (0.06) | -0.04 (0.07) | -0.12 (0.06) |
| PICEOL | -0.13 (0.04)* | -0.05 (0.04) | -0.04 (0.04) | -0.06 (0.05) | - | 0.61 (0.04) *** | -0.14 (0.07)*** |
| PUNGENOL | -0.13 (0.05)* | -0.08 (0.04) | -0.06 (0.04) | -0.04 (0.05) | 0.61 (0.03)*** | - | -0.63 (0.04)*** |
| PICEIN | 0.04 (0.05) | 0.05 (0.04) | 0.03 (0.04) | ^‡^NA | -0.16 (0.05)*** | -0.63 (0.03)*** | - |

^†^ See Table 1 for full description of traits.

^‡^ The model did not converge.

Level of statistical significance: **P* < 0.05; ***P* < 0.01; ****P* < 0.001.

Table S4. Fit of ABLUP and GBLUP models including additive effects only (A) and models including additive and dominance effects (A+D) as measured by the Akaike information criterion (AIC) and the Bayesian information criterion (BIC).

|  | AIC | | | |  | BIC | | | |
| --- | --- | --- | --- | --- | --- | --- | --- | --- | --- |
|  | A | AD | Δ* | Best model^‡^ |  | A | AD | Δ* | Best model^‡^ |
| **ABLUP**^†^ |  |  |  |  |  |  |  |  |  |
| HT | 13781.4 | 13776.9 | 4.50 | A+D |  | 13812.4 | 13813.0 | -0.66 | — |
| DBH | 9623.9 | 9623.2 | 0.71 | — |  | 9654.8 | 9659.3 | -4.44 | A |
| VOL | 4971.6 | 4971.1 | 0.49 | — |  | 5002.6 | 5007.2 | -4.67 | A |
| VELO | -633.7 | -633.0 | -0.74 | — |  | -607.8 | -602.7 | -5.07 | A |
| PICEOL | 572.9 | 574.9 | -2.00 | — |  | 599.0 | 605.4 | -6.36 | A |
| PUNGENOL | 587.8 | 589.6 | -1.76 | — |  | 614.0 | 620.1 | -6.12 | A |
| PICEIN | 800.0 | 802.0 | -2.00 | A |  | 826.2 | 832.5 | -6.30 | A |
|  |  |  |  |  |  |  |  |  |  |
| **GBLUP**^†^ |  |  |  |  |  |  |  |  |  |
| HT | 13773.7 | 13766.3 | 7.46 | A+D |  | 13804.7 | 13802.4 | 2.30 | A+D |
| DBH | 9616.9 | 9615.5 | 1.38 | — |  | 9647.8 | 9651.6 | -3.78 | A |
| VOL | 4965.0 | 4962.1 | 2.91 | A+D |  | 4995.9 | 4998.2 | -2.25 | A |
| VELO | -630.7 | -637.0 | 6.33 | A+D |  | -604.7 | -606.7 | 2.00 | — |
| PICEOL | 578.6 | 580.2 | -1.59 | — |  | 604.8 | 610.7 | -5.95 | A |
| PUNGENOL | 582.8 | 584.1 | -1.34 | — |  | 609.0 | 614.7 | -5.70 | A |
| PICEIN | 809.6 | 811.6 | -2.00 | A |  | 835.7 | 842.1 | -6.40 | A |

^†^ See Table 1 for full description of traits.

* Δ = difference between A and AD

^‡^The best model was determined as the model with the smallest value of AIC or BIC, with Δ > 2.





Figure S1. Heatmap of the realized additive genomic relationship matrix (***G***). The color key (top left corner) is represented as a histogram of pairwise G values. The red blocks (G ~ 0.5) near the diagonal represent full-sib families (i.e. individuals sharing both parents) and the off-diagonal light blue blocks (G ~ 0.25) represent half-sib families (i.e. individuals sharing one parent).





Figure S2. Heatmap of the realized dominance genomic relationship matrix ($\boldsymbol{D}$). The color key (top left corner) is represented as a histogram of pairwise D values. The light blue block (D ~ 0.25) near the diagonal represent full-sib crosses (i.e. individuals sharing both parents).

| (a) | 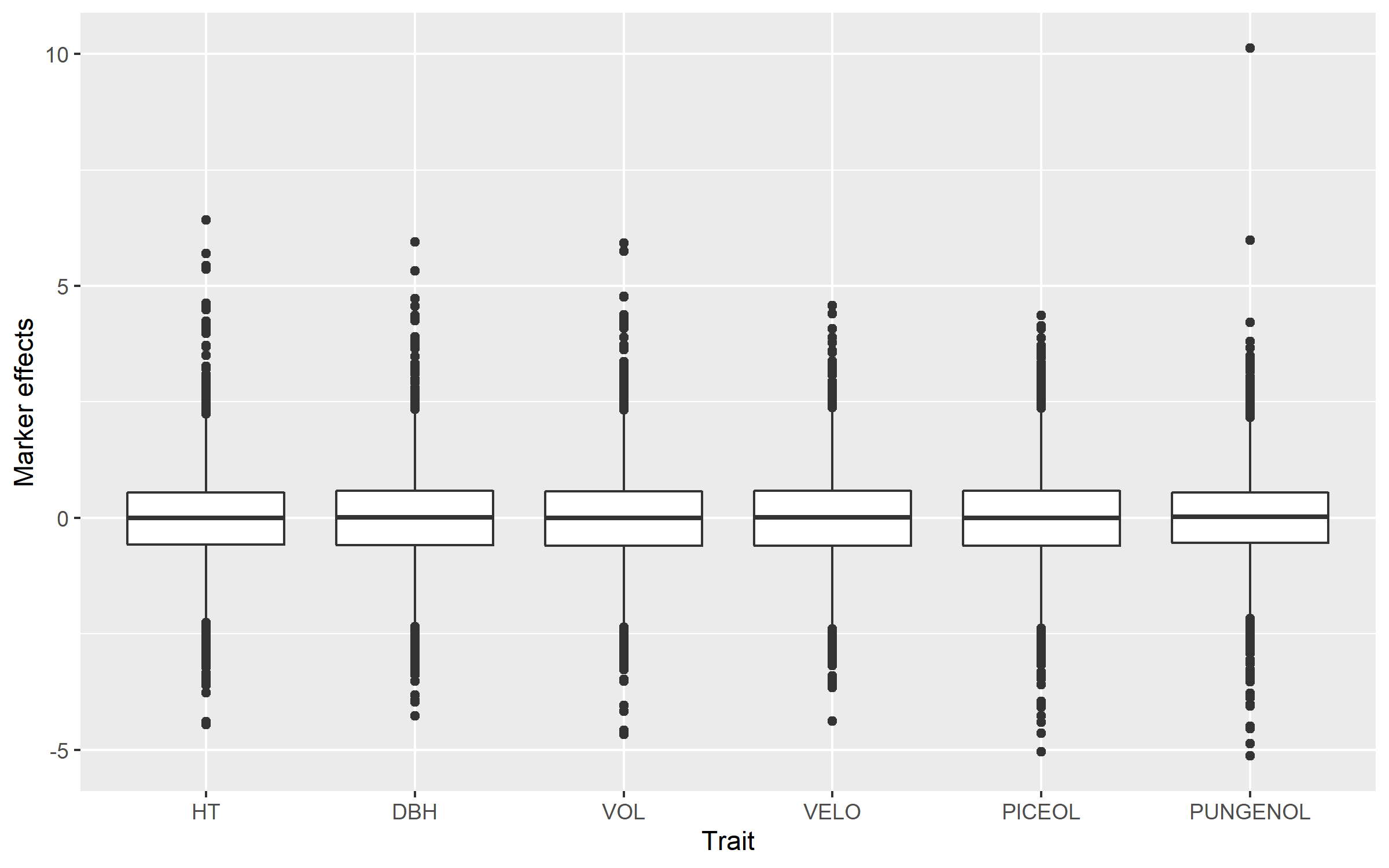 |
| --- | --- |
| (b) | 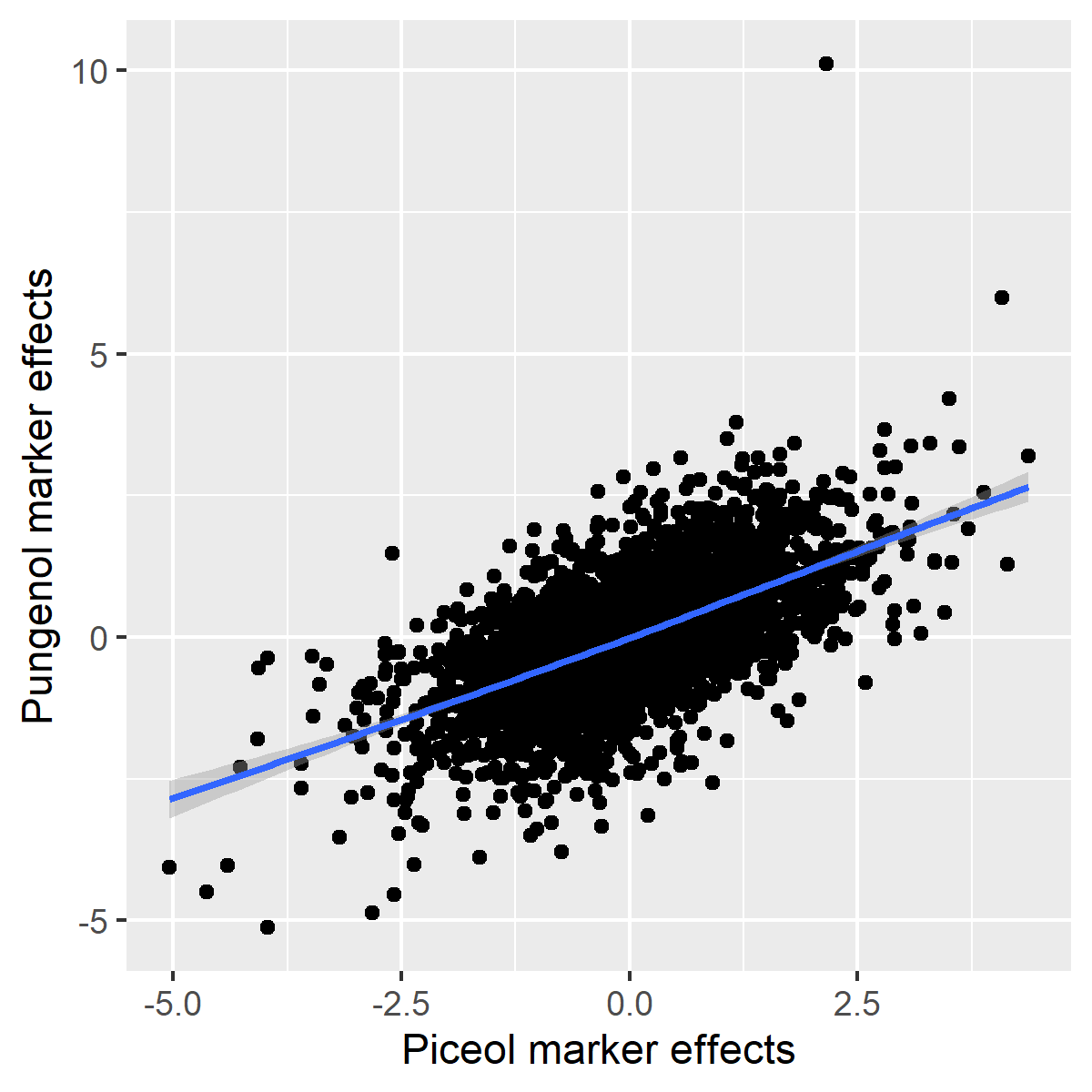 |

Figure S3. (a) Estimated additive marker effects from the BayesCπ additive-only model, and (b) correlation of marker effects between piceol and pungenol. For the purpose of this figure, marker effects for each traits were scaled to a mean of 0 and a variance of 1 for ease of visualization.

| (a) | 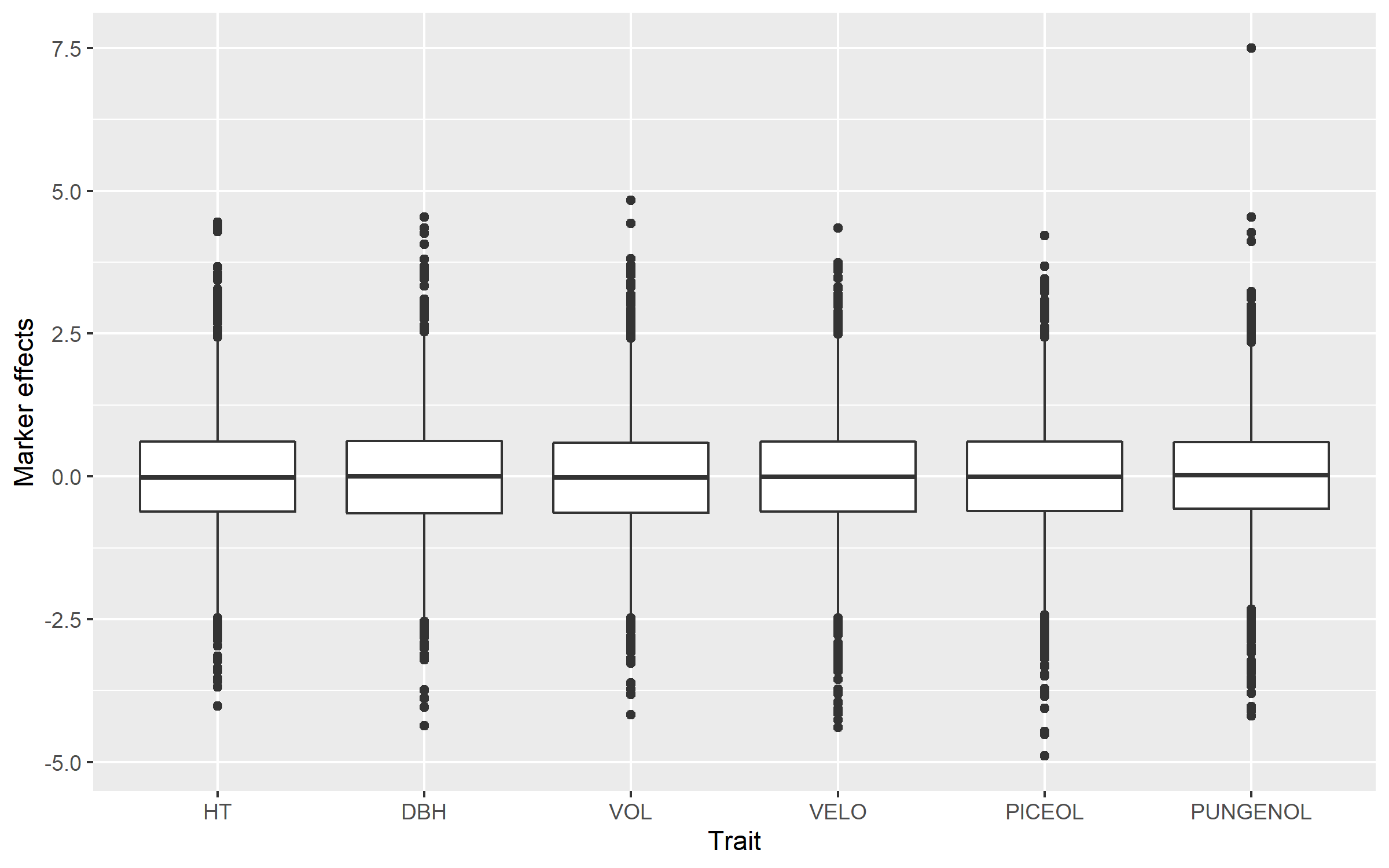 |
| --- | --- |
| (b) | 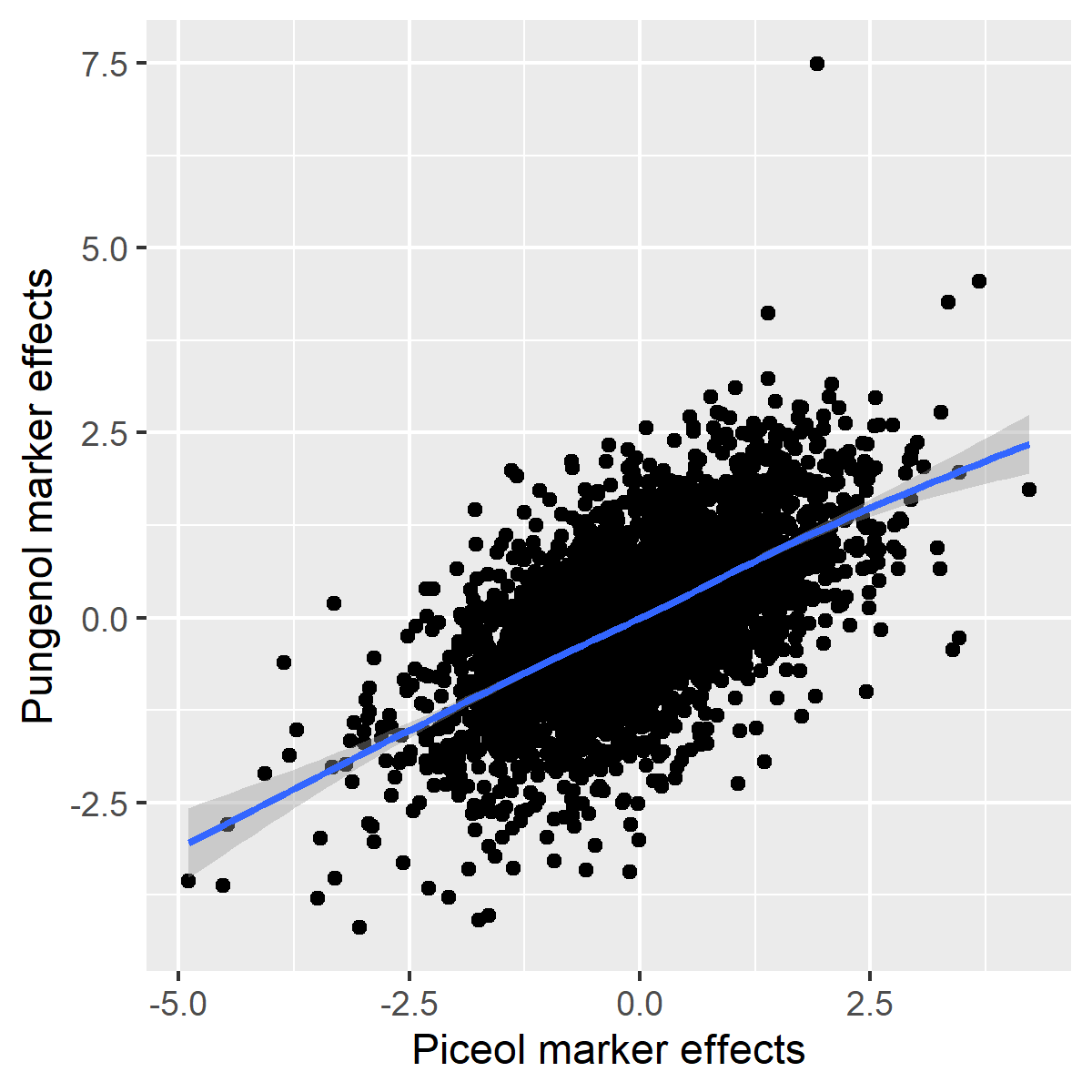 |

Figure S4. (a) Estimated additive + dominance marker effects from the BayesCπ additive-dominance model, and (b) correlation of marker effects between piceol and pungenol. For the purpose of this figure, marker effects for each traits were scaled to a mean of 0 and a variance of 1 for ease of visualization.

**Supplementary methods for the assessment of acetophenone aglycones (AAs)**

Quantitation accuracy of the extraction method was tested by spiking known amounts of AAs at the beginning of the extraction using needle tissue with aglycones concentrations below detection limits.

Control samples to check method reproducibility and instrument performance were routinely carried out throughout the study. Every 100 samples, one sample was extracted in triplicate to check the method technical variability. The standard deviation of the measurement obtained from our triplicate analysis was 5.5%, 5.2%, and 9.1% for picein, pungenol, and piceol, respectively. To monitor instrument performance, three samples were injected in every batch and the concentration of acetophenones used as a benchmark. Picein concentration was only used as a positive control for the presence of acetophenones in needle extracts, as this glycoside appears to be inactive against the spruce budworm in white spruce, contrary to the aglycones piceol and pungenol ([Mageroy et al., 2017](#_ENREF_33)). Thus, picein concentration was used as a benchmark in heritability and genetic correlation results, but the predictive ability of GS models and the expected genetic gains for this trait were not evaluated.
